# Ischaemia alters the effects of cardiomyocyte‐derived extracellular vesicles on macrophage activation

**DOI:** 10.1111/jcmm.14014

**Published:** 2018-12-04

**Authors:** Rafael Almeida Paiva, Tania Martins‐Marques, Katia Jesus, Teresa Ribeiro‐Rodrigues, Monica Zuzarte, Ana Silva, Liliana Reis, Maria da Silva, Paulo Pereira, Pieter Vader, Joost Petrus Gerardus Sluijter, Lino Gonçalves, Maria Teresa Cruz, Henrique Girao

**Affiliations:** ^1^ Coimbra Institute for Clinical and Biomedical Research (iCBR) Faculty of Medicine University of Coimbra Coimbra Portugal; ^2^ CNC.IBILI University of Coimbra Coimbra Portugal; ^3^ Faculty of Pharmacy University of Coimbra Coimbra Portugal; ^4^ Cardiology Department CHUC‐HG Coimbra Portugal; ^5^ Chronic Diseases Research Center (CEDOC) NOVA Medical School Faculdade de Ciências Médicas Universidade NOVA de Lisboa Lisboa Portugal; ^6^ Department of Experimental Cardiology University Medical Center Utrecht Utrecht The Netherlands; ^7^ Laboratory of Clinical Chemistry and Hematology University Medical Center Utrecht Utrecht The Netherlands; ^8^ Department of Cardiology Division of Heart & Lungs University Medical Center Utrecht Utrecht The Netherlands; ^9^ Interuniversity Cardiology Institute Netherlands (ICIN) Utrecht The Netherlands

**Keywords:** extracellular vesicles, intercellular communication, cardiomyocytes, macrophages, acute myocardial infarction

## Abstract

Myocardial ischaemia is associated with an exacerbated inflammatory response, as well as with a deregulation of intercellular communication systems. Macrophages have been implicated in the maintenance of heart homeostasis and in the progression and resolution of the ischaemic injury. Nevertheless, the mechanisms underlying the crosstalk between cardiomyocytes and macrophages remain largely underexplored. Extracellular vesicles (EVs) have emerged as key players of cell‐cell communication in cardiac health and disease. Hence, the main objective of this study was to characterize the impact of cardiomyocyte‐derived EVs upon macrophage activation. Results obtained demonstrate that EVs released by H9c2 cells induced a pro‐inflammatory profile in macrophages, via p38MAPK activation and increased expression of iNOS, IL‐1β and IL‐6, being these effects less pronounced with ischaemic EVs. EVs derived from neonatal cardiomyocytes, maintained either in control or ischaemia, induced a similar pattern of p38MAPK activation, expression of iNOS, IL‐1β, IL‐6, IL‐10 and TNFα. Importantly, adhesion of macrophages to fibronectin was enhanced by EVs released by cardiomyocytes under ischaemia, whereas phagocytic capacity and adhesion to cardiomyocytes were higher in macrophages incubated with control EVs. Additionally, serum‐circulating EVs isolated from human controls or acute myocardial infarction patients induce macrophage activation.

According to our model, in basal conditions, cardiomyocyte‐derived EVs maintain a macrophage profile that ensure heart homeostasis, whereas during ischaemia, this crosstalk is affected, likely impacting healing and post‐infarction remodelling.

## 1 INTRODUCTION

Acute myocardial infarction (AMI), due to a blockage of blood supply to the heart, restricts the availability to oxygen and nutrients, ultimately leading to cell death.^1^, ^2^ Although a fine‐tuned inflammatory response is essential for an efficient cardiac repair following injury, its abnormal intensification can be detrimental and it has been implicated in the pathological remodelling of the heart, namely in AMI and heart failure.^3^

It is acknowledged that a highly regulated intercellular communication network between cardiomyocytes, endothelial cells, fibroblasts and immune cells is vital to mount an adequate and efficient response during ischaemic wound healing.^2^, ^4^, ^5^ Accordingly, the release of pro‐inflammatory cytokines following cardiomyocyte death triggers the recruitment of leukocytes to the damaged sites.^6^ Macrophages are among the first responders, transmigrating across the vascular wall and infiltrating the infarcted area.^7^ Macrophages invade the cardiac tissue via interactions with extracellular matrix components, and further adhere to viable cardiomyocytes in the border zone, clearing cell debris and dead cells in the necrotic area.^8^ Concomitantly, fibroblasts acquire a pro‐inflammatory and matrix‐degrading phenotype, before suppression of inflammatory signals in transition to the proliferative phase, where macrophages secrete transforming growth factor (TGF)‐β and VEGF, contributing to myofibroblast transdifferentiation, fibrosis and angiogenesis.^9^, ^10^

It is well‐established that the role of macrophages goes beyond its canonical phagocytic function, being able to perform highly specialized, organ‐specific functions.^7^, ^11^, ^12^ Importantly, macrophages are recognized as one of the major cell populations of the heart, where they are implicated in the maintenance of homeostasis, cardiac regeneration in young mice, as well as in the progression and resolution of the ischaemic injury.^5^, ^13^, ^14^ Gene expression analysis of cardiac‐resident macrophages revealed that these cells display an organ‐specific phenotype, including increased expression of classical pro‐inflammatory interleukin (IL)‐1β and IL‐6, anti‐inflammatory IL‐10 and matrix metallopeptidase (MMP)13.^12^ In a recent study, macrophages were demonstrated to contribute to the electric impulse propagation between cardiomyocytes, through the distal atrioventricular (AV) node, in a Connexin (Cx)43‐dependent manner.^15^

Besides direct cell‐cell communication through gap junctions (GJ) and soluble factors‐mediated paracrine communication, extracellular vesicles (EVs) are important players in the crosstalk between cardiac cells.^16^, ^17^ Regarding their subcellular origin, EVs can be divided into exosomes, microvesicles or apoptotic bodies.^18^, ^19^ In each case, the mechanisms of EV secretion and uptake are highly regulated, and can be affected in response to various cellular stressors, including ischaemia.^17^ Mounting evidence has demonstrated that EVs are major conveyors of biological information, being able to deliver complex mixtures of cargo in a targeted manner, modulating the behaviour of receptor cells.^20^, ^21^, ^22^ In the heart, EVs secreted by adult mouse cardiomyocytes were shown to modulate gene expression in fibroblasts and activate endothelial cells.^20^, ^23^ EV secretion of heat shock protein (Hsp)60, a known activator of toll‐like receptor (TLR)4, by cardiomyocytes was reported to be upregulated in response to hypoxia.^24^ Recently, we showed that cardiomyocytes subjected to ischaemia release EVs that promote the growth of new vessels in the heart.^22^ Nevertheless, cardiomyocyte‐macrophage crosstalk via EVs remains elusive. In this study, we have been suggested that EVs secreted by cardiomyocytes are able to modulate macrophage function. Our results show for the first time that EVs participate in the communication between cardiomyocytes and macrophages, and that in pathological conditions, such as ischaemia, this crosstalk is affected, thus ascribing to EVs an important role in cardiomyocyte‐mediated regulation of macrophage activation.

## 2 MATERIALS AND METHODS

Detailed description of reagents and experimental procedures are listed in Data S1.

### 2.1 Human serum samples

Samples were obtained according to the Declaration of Helsinki (2008), with local research ethics committee approval (#CHUC‐057‐15) and written informed consent from all patients (Table 1). Serum samples were collected from AMI patients within 12 hours of admission to the Coronary Intensive Care Unit. Patients with no epicardial coronary artery disease were used as controls.

**Table 1:** Patient demographic data

	Control	AMI
n	12	15
STEMI	n/a	12 (80.0%)
M:F	6:6	9:6
Age (mean, SD)	57.3 ± 18.4	65.5 ± 11.9
CV risk		
Diabetes mellitus	4 (33.3%)	5 (33.3%)
Hypertension	8 (66.7%)	10 (66.7%)
Dyslipidemia	6 (50.0%)	14 (93.3%)
Smoker	3 (25.0%)	5 (33.3%)
Previous coronary artery disease	0	3 (20.0%)
Admission medication		
Aspirin	5 (41.7%)	14 (93.3%)
Ticagrelor/clopidogrel/prasugrel	0	12 (80.0%)
Oral anticoagulants	1 (8.3%)	1 (6.67%)
ACEI	7 (58.3%)	13 (86.7%)
β‐blocker	8 (66.7%)	12 (80.0%)
Spironolactone	0	2 (13.33%)
Statin	8 (66.7%)	15 (100.0%)

STEMI, ST‐segment elevation myocardial infarction; n, number; M, male; F, female; CV, cardiovascular; ACEI, angiotensin converting enzyme inhibitor.

### 2.2 EV isolation

EVs derived from cultured cells were isolated from conditioned medium after culturing in EV‐depleted medium or ischaemia‐mimetic solution for 2 hours, as previously described.^16^, ^25^, ^26^ Harvested supernatants were subjected to differential centrifugation at 4°C, starting with 300 *g*, 10 minutes, followed by 16.500 *g*, 20 minutes. Supernatants were filtered (0.22 μm) and ultracentrifuged at 120.000 *g*, 70 minutes. Circulating EVs were obtained as described previously.^25^ EVs were characterized by transmission electron microscopy (TEM) and nanoparticle tracking analysis (NTA). Unless stated otherwise, 1 × 10^6^ macrophages were stimulated, in EV‐depleted medium, with an average of 2.5 × 10^9^ cell‐derived EVs/mL or 3.75 μg/mL of human‐derived EVs.

### 2.3 Nitrite production assay

Nitrite levels were determined in cell supernatants using Griess reagent (1% sulphanilamide, 0.1% *N*‐1‐naphthylenediamine dihydrochloride and 2.5% phosphoric acid). Absorbance was measured at 550 nm with a Synergy HT multi‐mode microplate reader (BioTek, Bad Friedrichshall, Germany).

### 2.4 Trypsin resistance assay

A quantity of 1 mg/mL of trypsin was added to EVs for 10 minutes, at 37°C, to cleave membrane‐associated proteins. Protease action was inhibited by addition of EV‐depleted medium. EVs were filtered (0.22 μm) before incubation with recipient cells.

### 2.5 PKH26 dye uptake

EV labelling with PHK26 was performed according to manufacturer's instructions (PKH26 Fluorescent Cell Linker, Sigma‐Aldrich; St. Louis, MO, USA). Excess dye was washed by EV floatation on a discontinuous sucrose gradient (2.5‐0.4 mol/L), ultracentrifuged at 160.000 *g*, for 18 hours.^16^ EV‐containing fractions (four fractions, densities 1.08‐1.2 g/mL) were collected and washed with PBS by ultracentrifugation. Raw 264.7 cells, grown on glass coverslips, were stained with Alexa Fluor 488‐conjugated wheat germ agglutinin (WGA) for 10 minutes, at 37°C, and washed with PBS, before incubation with fluorescently labelled EVs for 30 minutes. Cells were fixed with 4% paraformaldehyde (PFA), followed by nuclei staining with 4′,6‐diamidino‐2‐phenylindole (DAPI). Samples were mounted in Mowiol 4‐88 reagent. Images were collected with an Axio Observer.Z1 (Carl Zeiss AG, Jena, Germany).

### 2.6 Adhesion assays

For cell‐cell adhesion studies, H9c2 cells or primary cardiomyocytes were cultured in coverslips until confluency was reached. In cell‐matrix experiments, coverslips were coated with fibronectin (5 μg/mL, 30 minutes at 37°C), collagen I (50 μg/mL) or collagen IV (50 μg/mL), for 2 hours at room temperature. Raw 264.7 or peritoneal macrophage suspensions were labelled with 1.5 μmol/L Calcein‐AM (Thermo Fisher Scientific, Waltham, MA, USA) for 30 minutes, at 37°C. Excess of Calcein was removed by washing with PBS. Labelled macrophages were incubated with H9c2 cells or the indicated matrices for 1 hour, at 37°C, followed by fixation with 4% PFA. Images were acquired in a Axio Observer.Z1 and analysed with ImageJ (National Institutes of Health, NIH).

### 2.7 Phagocytosis assay

Latex beads (3.9 μm diameter, Life Technologies) were opsonized with goat serum (5%, v/v) for 1 hour at room temperature. Opsonized beads were incubated with the macrophages for 20 minutes, at 37°C (10 beads/cell). Non‐internalized particles were extensively washed out with PBS, after which cells were fixed with 4% PFA. To identify adherent opsonized beads that were not internalized, cells were incubated for 45 minutes with Alexa Fluor donkey anti‐goat 488 antibody, together with actin staining with Rhodamine‐Phalloidin (Sigma‐Aldrich). Images were acquired in a Axio Observer.Z1 and analysed with ImageJ.

### 2.8 Statistical analysis

All data represent at least three independent experiments and are expressed as individual data points with mean. Data were analysed with GraphPad Prism 6, version 6.01 (GraphPad Software, Inc.). Repeated measure ANOVA, followed by Tukey's post hoc test or two‐tailed unpaired Student's *t* test with Bonferroni correction was applied.

## 3 RESULTS

### 3.1 Characterization of cardiomyocyte‐derived EVs

**Figure 1:**
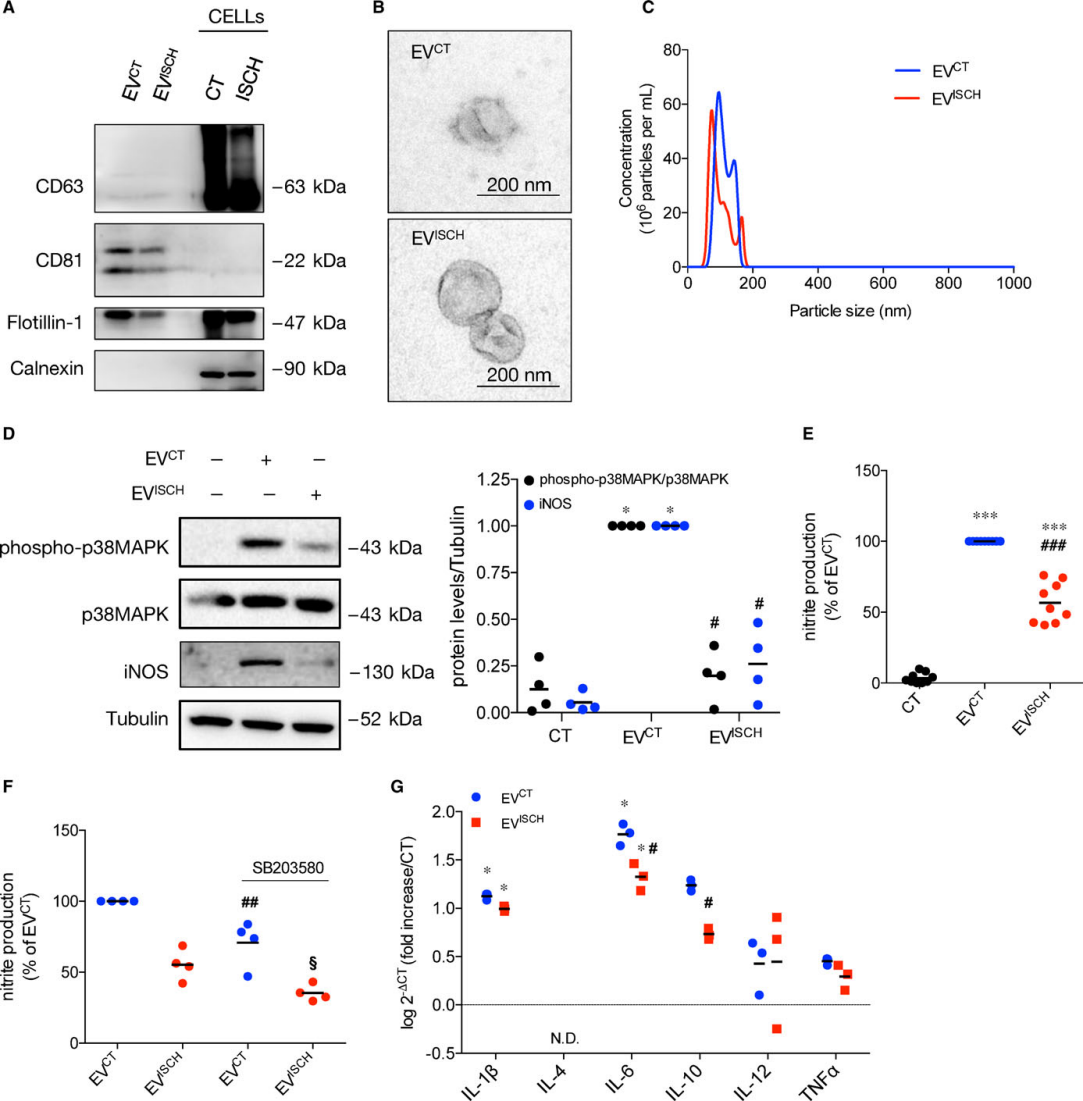
Cardiomyocyte‐derived EVs modulate activation profile of macrophages. The cardiomyoblast cell line H9c2 was cultured under control (EV^CT^) or simulated ischaemia (EV^ISCH^) for 2 hours, after which EVs were isolated by differential centrifugation. (A) The protein profile of EVs and cellular (CELLs) extracts was evaluated by WB, loading 20 μg of total protein in each case. (B) Representative image of EV^CT^ and EV^ISCH^ observed by TEM. (C) Representative NTA analysis of concentration and size of EVs derived from 5.5 × 10^6^ H9c2 cells, maintained either in control or ischaemia for 2 hours. (D) Macrophages were incubated with EV^CT^ or EV^ISCH^ for 24 hours. p38MAPK phosphorylation and iNOS expression were evaluated by WB in macrophages after incubation with EV^CT^ or EV^ISCH^ for 24 hours. Graph depicts WB quantification. *P < 0.05 vs CT, ^#^ *P* < 0.05 vs EV^CT^ (n = 4). (E) Nitrite production was determined using the Griess reagent assay in macrophages stimulated with EV^CT^ or EV^ISCH^ for 24 hours. Results are expressed as percentage of nitrite production over macrophages treated with EV^CT^. *^**^ *P* < 0.001 vs CT, ^###^ *P* < 0.001 vs EV^CT^ (n = 9). (F) Nitrite production was determined in macrophages, was pre‐treated with 10 μmol/L SB203580 for 1 hour, before incubation with EV^CT^ or EV^ISCH^ for 24 hours. Results are expressed as percentage of nitrite production over macrophages treated with EV^CT^. ^##^ *P* < 0.01 vs EV^CT^, ^§^ *P* < 0.05 vs EV^ISCH^ (n = 4). (G) mRNA expression levels of IL‐1β, IL‐4, IL‐6, IL‐10, IL‐12 and TNFα were assessed by RT‐qPCR in macrophages stimulated with EV^CT^ or EV^ISCH^. Results were normalized using GAPDH and expressed relatively to naïve macrophages (CT). Values are expressed as log^2 − ΔCT^. N.D. not detected. *P < 0.05 vs CT, ^#^ *P* < 0.05 vs EV^CT^ (n = 3)

We started by characterizing, in terms of protein content, size and morphology, the population of EVs secreted by the myoblast cell line H9c2, widely used as a cardiomyocyte model, maintained either in control (EV^CT^) or ischaemia‐mimetic conditions (EV^ISCH^).^27^, ^28^ Our results demonstrate that the vesicle extract was positive for canonical EV markers, CD63, CD81 and Flotillin‐1, and was devoid of Calnexin, an endoplasmic reticulum protein that is expected to be absent from EVs (Figure 1A, Supporting Information Figure S1A). No significant differences were found either in morphology of EV^CT^ and EV^ISCH^, assessed by TEM, which confirmed the presence of cup‐shaped vesicles (Figure 1B),^25^, ^29^ or in particle size (50‐200 nm) and concentration (2.89 ± 0.012 × 10^9^ particles/mL of EV^CT^, modal size of 95.2 nm vs 2.74 ± 0.012 × 10^9^ particles/mL of EV^ISCH^, modal size of 73.7 nm; Figure 1C), evaluated by NTA.^19^ To validate our data, we isolated EVs from neonatal rat ventricular myocytes (NRVM), cultured in control (NRVM_EV^CT^) or subjected to ischaemia (NRVM_EV^ISCH^). Similarly, no major differences were found regarding EV markers (Supporting Information Figure S1B).

### 3.2 Cardiomyocyte‐derived EVs modulate the activation profile of macrophages

Since cardiac ischaemia triggers a strong pro‐inflammatory response, we have been suggested that EVs released by cardiomyocytes during ischaemia evoke macrophage activation.^2^, ^11^ To address this, we compared the macrophage response elicited by EVs derived from H9c2 cells maintained either in control or simulated ischaemia. Given its association with cardioprotection, we evaluated the effect of EVs on activation of p38 mitogen‐activated protein kinases (p38MAPK), inducible nitric oxide synthase (iNOS) expression and nitrite production by macrophages.^30^, ^31^ Results in Figure 1D,E show that macrophages challenged with EV^ISCH^ for 24 hours presented a significantly lower increase in p38MAPK phosphorylation, iNOS expression and nitrite production, when compared with macrophages incubated with EV^CT^. Nonetheless, when compared with naïve macrophages, both EV^CT^ and EV^ISCH^ were able to increase all activation parameters, suggesting that EVs derived from cardiomyocytes trigger a strong activation of macrophages. In accordance, a trend towards an increase in the levels of phosphorylation of the p65 subunit of inducible nuclear factor‐κB (NF‐κB) was found after challenging with EV^CT^ (Supporting Information Figure S1C). Pharmacological inhibition of the p38MAPK pathway reduced EV‐induced nitrite production by macrophages, showing that p38MAPK activation is responsible, at least partially, for the observed increase in nitrite production (Figure 1F). To validate these results, we performed experiments using isolated murine peritoneal macrophages, whose purity was confirmed by CD11b staining (Supporting Information Figure S1D). Similar to the observed with the macrophage cell line, our data show that stimulation with EV^CT^ enhanced iNOS expression, p38MAPK and p65 phosphorylation in primary macrophages, an effect that was less pronounced in cells treated with EV^ISCH^ (Supporting Information Figure S1E,F).

Next, we assessed the impact of EVs on the expression of inflammation‐associated genes. Incubation with either EV^CT^ or EV^ISCH^ resulted in increased mRNA expression of IL‐1β and IL‐6, when compared with unprimed macrophages. Nevertheless, we found that EV^ISCH^ had significantly less effect on the production of IL‐6 and IL‐10, when compared with EV^CT^ (Figure 1G).

When macrophages were stimulated with NRVM‐derived EVs, both NRVM_EV^CT^ and NRVM_EV^ISCH^ increased p38MAPK and NF‐κB/p65 activation, iNOS expression, nitrite production, mRNA levels of IL‐1β, IL‐6, IL‐10 and tumour necrosis factor (TNF)α and attenuated the production of IL‐12 (Supporting Information Figure S2A‐D), in similar patterns.

To exclude confounding factors, we evaluated the effect of ischaemia on the viability of H9c2 cells, as well as the effect of cardiomyocyte‐derived EVs on the viability of macrophages. Our data showed that, in these experimental conditions, viability in both cell types was not significantly affected (Supporting Information Figure S2E,F).

### 3.3 Vesicle surface proteins are implicated in macrophage activation induced by EVs from ischaemic cardiomyocytes

**Figure 2:**
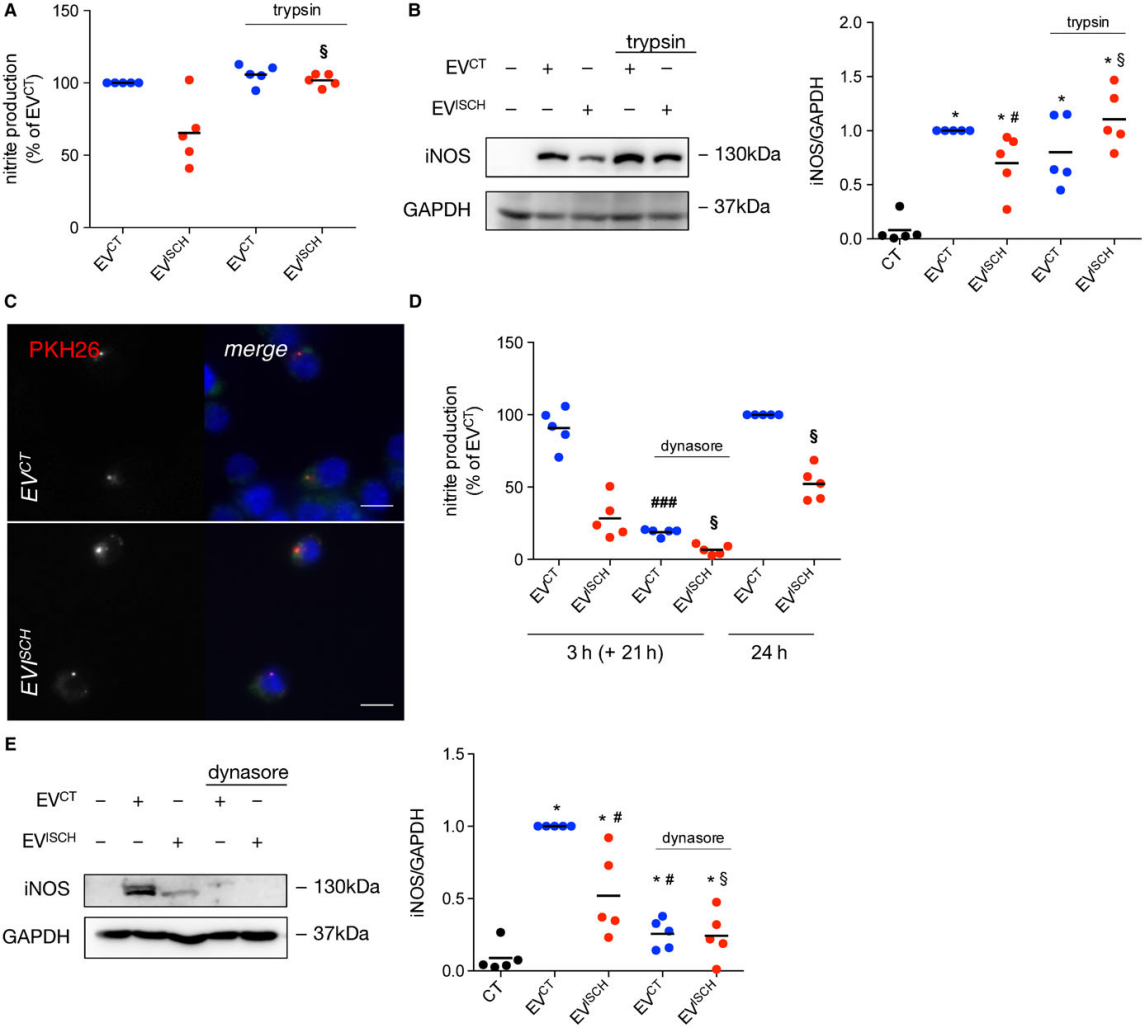
Vesicle surface proteins are implicated in macrophage activation induced by EVs from ischaemic cardiomyocytes. (A) EV^CT^ or EV^ISCH^ were treated with 1 mg/mL trypsin for 10 minutes. Trypsin‐treated or untreated EVs were further incubated with macrophages for 24 hours. Nitrite production was determined and results are expressed as percentage of nitrite production over macrophages treated with EV^CT^. ^§^ *P* < 0.05 vs EV^ISCH^ (n = 4). (B) iNOS expression was evaluated in macrophages challenged with trypsin‐treated or untreated EV^CT^ or EV^ISCH^ for 24 hours, by WB. Graph depicts quantification levels. *P < 0.05 vs CT, ^#^ *P* < 0.05 vs EV^CT^, ^§^ *P* < 0.05 vs EV^ISCH^ (n = 5). (C) WGA‐stained macrophages (green) were incubated with PKH26‐labelled EV^CT^ or EV^ISCH^ (red) for 30 minutes, after which cells were fixed and visualized by fluorescence microscopy. Nuclei were stained with DAPI (blue). Scale bars 5 μm. (D) Macrophages were treated with 80 μmol/L dynasore for 30 minutes, followed by incubation with EV^CT^ or EV^ISCH^ for 3 hours. Medium was then replaced with EV‐depleted medium and cells were kept in culture for an additional 21 hours. Nitrite production was determined and results are expressed as percentage of nitrite production over macrophages treated with EV^CT^ for 24 hours. Cells challenged with EV^CT^ or EV^ISCH^ for 24 hours were used as control. ^###^ *P* < 0.001 vs EV^CT^, ^§^ *P* < 0.05 vs EV^ISCH^ (n = 5). (E) iNOS expression was evaluated in macrophages either treated or not with dynasore, followed by stimulation with EV^CT^ or EV^ISCH^, by WB. Graph depicts quantification levels. *P < 0.05 vs CT, ^#^ *P* < 0.05 vs EV^CT^, ^§^ *P* < 0.05 vs EV^ISCH^ (n = 5)

EVs can interact and be internalized by cells through distinct mechanisms that are likely to affect their biological and functional impact.^16^, ^32^ To evaluate whether the effects of cardiomyocyte‐released EVs on macrophages rely on EV surface proteins, we performed trypsin resistance assays, aiming at digesting proteins exposed to the extraluminal space, without affecting intraluminal cargo.^16^ Results in Figure 2A,B show that trypsin treatment of EV^CT^ did not have a significant effect on nitrite production nor iNOS expression in macrophages. However, when surface proteins of EV^ISCH^ were cleaved with trypsin, the levels of both nitrites and iNOS increased, when compared with non‐treated EV^ISCH^ (Figure 2A,B), suggesting that inhibitory proteins are located at the surface of EV^ISCH^, contributing for the impaired macrophage response.

Besides juxtacrine signalling, EVs can affect target cells after phagocytosis, endocytosis and/or fusion with the plasma membrane.^33^, ^34^ To evaluate whether macrophages are able to internalize cardiomyocyte‐derived EVs, we started by staining EV^CT^ and EV^ISCH^ with PKH26, after which we assessed the presence of fluorescently labelled EVs inside macrophages. Both EV^CT^ and EV^ISCH^ were internalized by macrophages (Figure 2C), with no obvious differences between the two populations. To further explore the involvement on EV endocytosis upon macrophage response, macrophages were pre‐treated with the endocytic inhibitor dynasore for 30 minutes, followed by incubation with EVs for 3 hours. Nitrite production and iNOS expression were evaluated 21 hours after the stimuli. Results in Figure 2D,E showed that dynasore abrogated the effect of both control and ischaemic EVs, suggesting that EVs have to be taken up to exert their effects. Interestingly, when comparing the levels of nitrite production after incubation with EVs for 3 or 24 hours, the effects elicited by EV^CT^ were similar, regardless of the incubation time. However, the effect of EV^ISCH^ on nitrite production by macrophages was intensified over time, suggesting that EV^ISCH^ are internalized with slower kinetics (Figure 2D). Altogether, these results suggest that proteins present at the surface of EV^ISCH^ either reduce their tropism towards macrophages, or negatively affect the interaction with the cellular machinery required for the docking and/or EVs uptake, delaying their internalization rate. Alternatively, surface proteins of EV^ISCH^ may act as repressors of intracellular pathways related with nitrite production and iNOS expression.

### 3.4 Cardiomyocyte‐derived EVs modulate macrophage adhesion to cardiomyocytes and macrophage‐matrix adhesion

**Figure 3:**
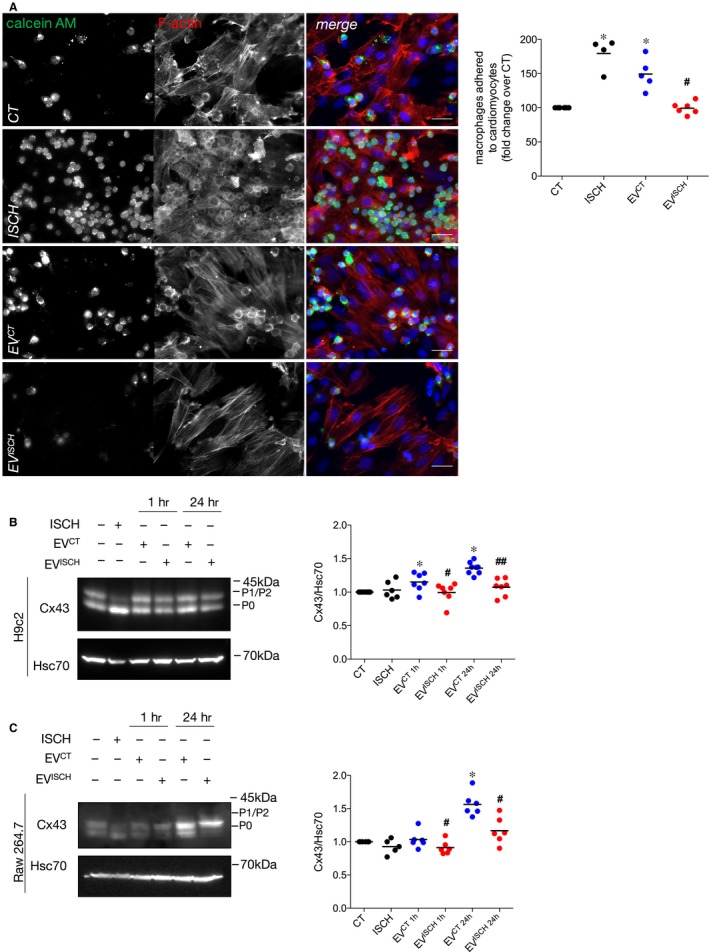
Control EVs increase macrophage adhesion to cardiomyocytes. (A) Macrophages were labelled with 1.5 μmol/L calcein‐AM (green) and further incubated with previously adherent H9c2 cells for 1 hour. Where indicated, EV^CT^ or EV^ISCH^ were added or simulated ischaemia was performed for 1 hour. F‐actin was stained with Rhodamine‐Phalloidin (red), and nuclei were stained with DAPI (blue). Scale bars 20 μm. Graph depicts the number of adherent macrophages. *P < 0.05 vs CT, ^#^ *P* < 0.05 vs EV^CT^ (*n* = 4‐6). (B) H9c2 cells were incubated with EV^CT^ or EV^ISCH^ for 1 or 24 hours. Cx43 levels were analysed by WB. Hsc70 was used as loading control. Graph depicts normalized levels of Cx43. *P < 0.05 vs CT, ^#^ *P* < 0.05, ^##^ *P* < 0.01 vs EV^CT^ (n = 6‐7). (C) Raw 264.7 cells were incubated with EV^CT^ or EV^ISCH^ for 1 or 24 hours. Graph depicts normalized levels of Cx43. *P < 0.05 vs CT, ^#^ *P* < 0.05 vs EV^CT^ (*n* = 5‐6)

Following myocardial ischaemia, induction of several chemokines and cytokines promotes the recruitment of inflammatory cells to the injured sites. Therefore, we sought to address whether EVs affected the adhesion of macrophages to cardiomyocytes. Results in Figure 3A show that the number of macrophages that adhered to cardiomyocytes increased in ischaemia. When adhesion assays were performed in the presence of H9c2‐derived EV^CT^, adhesion of Raw 264.7 and primary macrophages to cardiomyocytes was enhanced, whereas EV^ISCH^ had no effect, when compared with control cells (Figure 3A and Supporting Information Figure S3A). Experiments carried out with NRVM‐derived EVs further confirmed these results (Supporting Information Figure S3B).

It was previously shown that Cx43‐mediated intercellular communication between cardiomyocytes and macrophages is important for the electrical impulse propagation in the heart.^15^ Besides its major role on GJ communication, Cx43 has been reported to participate in cell‐cell adhesion.^35^ Thus, we next investigated whether the changes observed in cardiomyocyte‐macrophage adhesion were associated with alterations on Cx43. Our results showed that, both in cardiomyocytes and macrophages, Cx43 levels increased after 24 hours of stimulation with H9c2‐derived EV^CT^ (Figure 3B,C), when compared with cells maintained in the absence of EVs. Also, when comparing EV^CT^ with EV^ISCH^, both macrophages and H9c2 cells presented higher levels of Cx43, which could partially explain the increased adhesion in the presence of EV^CT^. However, when incubated with EVs secreted by NRVM, the levels of Cx43 were similar in macrophages stimulated with NRVM_EV^CT^ or NRVM_EV^ISCH^ (Supporting Information Figure S4A). When subjected to ischaemia, despite no differences were found in total levels, there was a striking accumulation of dephosphorylated Cx43 (P0 band in the WB), in both cardiomyocytes and macrophages, suggesting that the increased adhesion observed in ischaemia (Figure 3A) is associated not only with EV‐driven mechanisms, but also with a subcellular redistribution and/or post‐translational modifications on Cx43.

To establish a causal relationship between adhesion of macrophages and Cx43 levels, we looked for the existence of GJ between these two cell types. The functionality of these GJ was assessed by a dye transfer assay, in which calcein‐loaded macrophages were co‐cultured with H9c2 cells. The presence of calcein in H9c2 cells 1 hour after co‐culture, demonstrated that macrophages and cardiomyocytes can form functional GJ, allowing the exchange of information between these cells (Supporting Information Figure S4B), which can be blocked by heptanol treatment. Moreover, the presence of Cx43 in contacts between macrophages and cardiomyocytes in mouse hearts reinforced the hypothesis that Cx43 mediates the interaction between these two populations of cardiac cells (Supporting Information Figure S4C).

**Figure 4:**
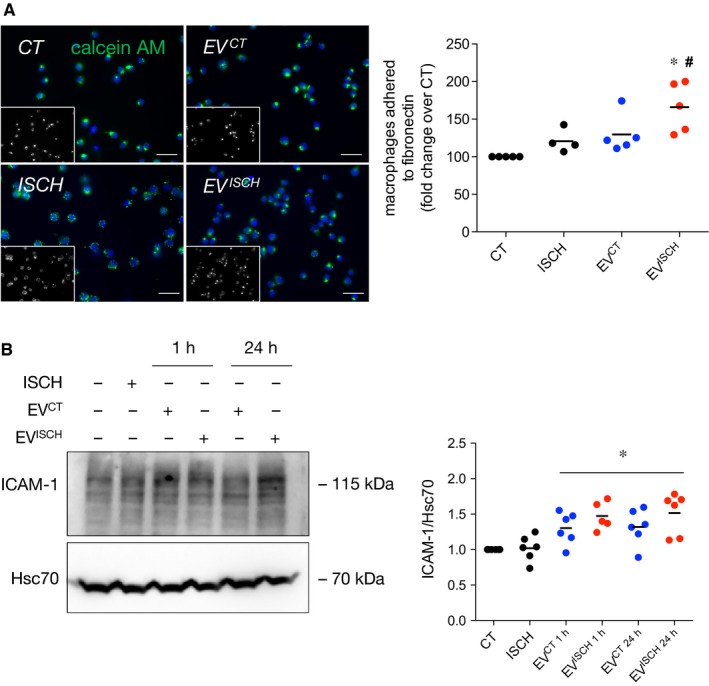
Ischaemic EVs increase adhesion of macrophages to a fibronectin matrix. (A) Macrophages were labelled with 1.5 μmol/L Calcein‐AM (green) and added on top of a fibronectin‐based matrix. Where indicated, EV^CT^ or EV^ISCH^ were added or simulated ischaemia was performed for 1 hour. Nuclei were stained with DAPI (blue). Scale bars 20 μm. Graph depicts the number of adherent macrophages. *P < 0.05 vs CT, ^#^ *P* < 0.05 vs EV^CT^ (n = 4‐5). (B) Macrophages were incubated with cardiomyocyte‐derived EV^CT^ or EV^ISCH^ for 1 or 24 hours. Graph depicts the levels of ICAM‐1. *P < 0.05 vs CT (n = 5‐6)

As extracellular matrix proteins are important to regulate and transduce inflammatory signalling after ischaemia, we proceeded to evaluate whether cardiomyocyte‐derived EVs affected macrophage‐matrix adhesion.^36^ Our results show that EV^ISCH^ enhanced the adhesion of both Raw 264.7 cells and primary macrophages to a fibronectin‐based matrix, when compared with naïve macrophages, or macrophages stimulated with EV^CT^ (Figure 4A, Supporting Information Figure S5A,B). Concomitantly, an increased expression of intercellular adhesion molecule 1 (ICAM‐1) was observed in macrophages exposed to H9c2‐derived EV^CT^ and EV^ISCH^, for 1 or 24 hours (Figure 4B), suggesting a correlation between increased expression of ICAM‐1 and cell‐matrix adhesion. Adhesion of macrophages to collagen I or IV‐based matrices was not significantly altered (Supporting Information Figure S6A,B).

### 3.5 Cardiomyocyte‐derived EVs increase macrophage phagocytic activity and resistance to oxidative damage

**Figure 5:**
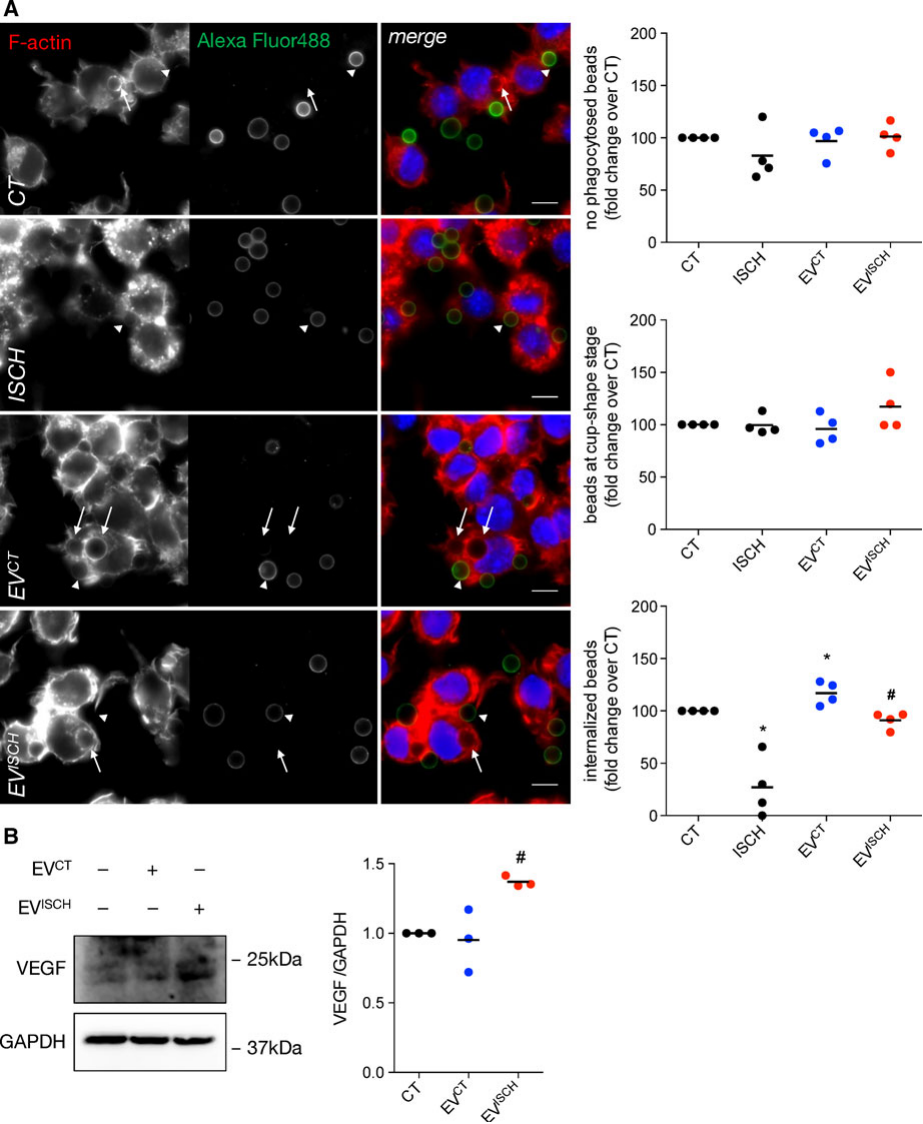
Control EVs increase the phagocytic capacity of macrophages. (A) Macrophages were incubated with opsonized latex beads for 20 minutes (ratio of 10 beads/cell). Non‐internalized beads were labelled with Alexa Fluor 488 antibodies (green). F‐actin was stained with Rhodamine‐Phalloidin (red). Graphs depict the total number of beads/phagocytic macrophages, represented as percentage of fold change over control (CT), the number of beads within cup‐shaped unsealed nascent phagosomes (positive Alexa Fluor 488 staining, arrow heads) and the number of beads in sealed phagosomes (negative Alexa Fluor 488 staining, arrows). Nuclei were stained with DAPI (blue). Scale bars 10 μm. *P < 0.05 vs CT, ^#^ *P* < 0.05 vs EV^CT^ (n = 4).(B) Macrophages were incubated with EV^CT^ or EV^ISCH^, for 24 hours. Graph depicts quantification of VEGF levels analysed by WB. ^#^ *P *< 0.05 vs EV^CT^ (n = 3)

Phagocytosis of dead cells and matrix debris occurs as part of the inflammatory phase after acute myocardial ischaemia.^2^ To address the effect of cardiomyocyte‐derived EVs upon phagocytic activity of macrophages, we evaluated, in Raw 264.7 cells and primary peritoneal macrophages, the (a) total number of phagocytosed opsonized latex beads, (b) number of beads at early stages of phagocytic cup formation (Figure 5A, arrow heads), and (c) number of beads within sealed phagosomes (internalized beads, Figure 5A, arrows). Although the total number of phagocytosed beads was not significantly affected, the number of beads within sealed phagosomes was higher in macrophages stimulated with control‐derived EVs, when compared with unstimulated cells, or with cells incubated with EV^ISCH^ (Figure 5A, Supporting Information Figure S7A and Figure S8A). Given that degradation of engulfed material occurs only after phagosome enclosure, this suggests that EV^CT^ increase the rate and/or efficiency of phagocytosis, whereas EV^ISCH^ did not affect phagocytic activity. On the other hand, cells subjected to ischaemia presented a marked decrease in the number of internalized beads that can be ascribed to decreased cellular ATP levels (Supporting Information Figure S8B).

Increased VEGF secretion by macrophages in the heart has been associated with ischaemic wound healing and angiogenesis.^10^, ^37^ Results in Figure 5B and Supporting Information Figure S8C show that the levels of VEGF increased in macrophages incubated with EV^ISCH^, whereas EV^CT^ did not induce a significant effect, suggesting that the repair profile of macrophages in ischaemia can be modulated by EVs secreted by cardiomyocytes.

We next sought to clarify whether the differential phenotypical alterations on macrophages induced by EVs can confer protection or contribute to tissue damage. For that, we subjected H9c2 cells to 1 hour of ischaemia, followed by 30 minutes of reperfusion (*I*/*R*), in the presence or absence of either naïve macrophages (Mϕ), or macrophages primed with EV^CT^ or EV^ISCH^ for 24 hours (Mϕ + EV^CT^ or Mϕ + EV^ISCH^ respectively). The levels of the lipid peroxidation marker, 4‐hydroxynonenal (4‐HNE), were used as a readout of oxidative stress caused by *I*/*R* stimuli. Expectedly, *I*/*R* induced an increase in the levels of 4‐HNE in cardiomyocytes (Supporting Information Figure S9), when compared with controls. More importantly, the oxidative damage caused by *I*/*R* was prevented in cells incubated with Mϕ + EV^CT^, but not with naïve macrophages, nor with EV^ISCH^—primed macrophages (Supporting Information Figure S9), suggesting that EV^ISCH^ no longer have the ability to prevent oxidative damage caused by *I*/*R*.

### 3.6 Circulating human EVs modulate the activation profile of macrophages

**Figure 6:**
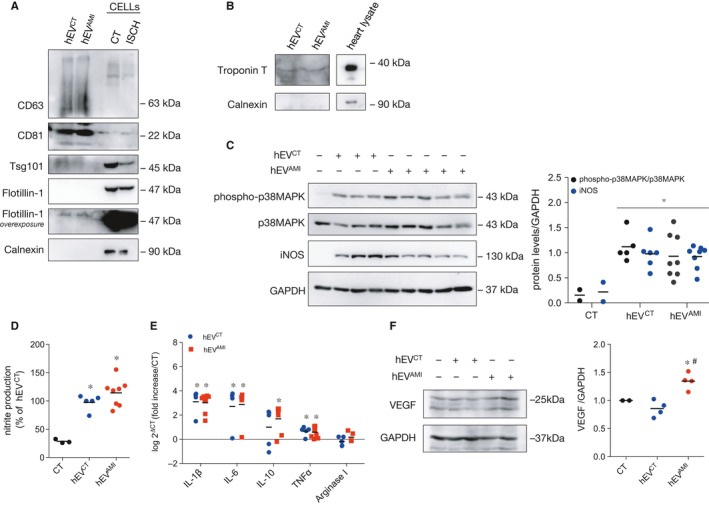
Circulating human EVs modulate the activation profile of macrophages. Circulating human EVs were isolated from serum of controls (hEV^CT^) or AMI patients (hEV^AMI^).(A) EV protein profile was evaluated by WB. H9c2 cell extracts (CELLs) were used as control. Twenty microgram of total protein was loaded in each case. (B) Representative WB analysis of circulating EVs. Troponin T was used as a cardiomyocyte marker and Calnexin was used as a negative EV marker. Heart lysates were used as positive control. (C) Macrophages were incubated with hEV^CT^ or hEV^AMI^ for 24 hours. p38MAPK phosphorylation and iNOS expression were evaluated by WB. Graph depicts WB quantification. *P < 0.05 vs CT (n = 5‐8). (d) Nitrite production was determined in macrophages treated with hEV^CT^ or hEV^AMI^ for 24 hours. Results are expressed as percentage of nitrite production over macrophages treated with hEV^CT^. *P < 0.05 vs CT (n = 5‐8). (E) mRNA expression levels of IL‐1β, IL‐6, IL‐10, TNFα and Arginase I was assessed using RT‐qPCR after treatment with hEV^CT^ or hEV^AMI^ for 24 hours. Results were normalized using GAPDH and expressed relatively to naïve macrophages (CT). Values are expressed as log^2 − ΔCT^ (n = 5‐8). (f) VEGF levels were evaluated in macrophages stimulated with hEV^CT^ or hEV^AMI^ for 24 hours. Graph depicts quantification data. *P < 0.05 vs CT, ^#^ *P* < 0.05 vs hEV^CT^ (n = 4)

To extend the biological relevance of our findings to a human pathophysiological context, we assessed the macrophage response elicited by circulating EVs derived from human serum of AMI patients (hEV^AMI^), or controls (hEV^CT^), without epicardial coronary artery disease. First, we characterized the circulating vesicles, showing that they were enriched in EV markers, including the tetraspanins CD63 and CD81, Tumour susceptibility gene 101 protein (Tsg101) and Flotillin‐1 and were devoid of Calnexin (Figure 6A). Strikingly, vesicles isolated from human serum were also positive for the cardiomyocyte marker Troponin T, indicating that, at least part of these EVs are from cardiac origin (Figure 6A). Moreover, circulating vesicles presented the typical EV morphology and size (Supporting Information Figure S10A), with no major differences among vesicles isolated from controls or AMI patients. NTA analysis (Supporting Information Figure S10B) showed that the number of EVs recovered from AMI patients was higher than in controls (3.34 ± 0.012 × 10^8^ particles/mL of hEV^AMI^, and 2.32 ± 0.08 × 10^8^ particles/mL of hEV^CT^) and slightly larger (modal size of 120.0 nm in hEV^AMI^, and 93.1 nm in hEV^CT^), as we have seen before.^38^

Next, we evaluated the effect of circulating EVs on macrophages. In accordance with the results obtained with cardiomyocyte‐derived EVs, our data demonstrate that both hEV^CT^ and hEV^AMI^ induced iNOS expression, p38MAPK and NF‐κB/p65 activation and nitrite production by macrophages (Figure 6C,D, Supporting Information Figure S10C), as well a higher production of IL‐1β, IL‐6, IL‐10 and TNFα (Figure 6E), comparing with naïve macrophages. However, no significant differences were found when comparing the effects of hEV^CT^ and hEV^AMI^.

**Figure 7:**
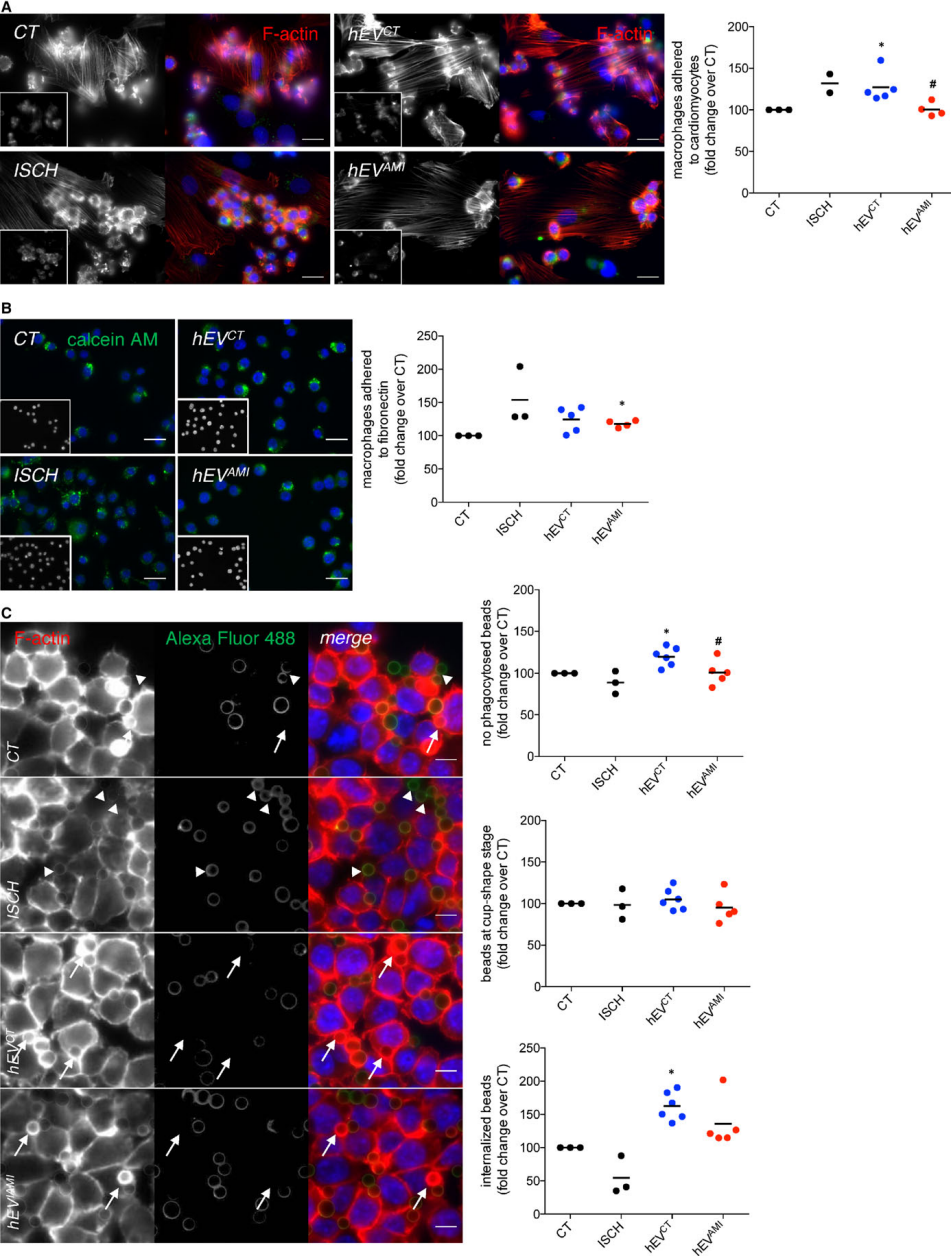
Circulating human EVs modulate cell‐cell, cell‐matrix adhesion and phagocytic activity of macrophages. (A) Macrophages were labelled with 1.5 μmol/L calcein‐AM (insets; green) and further incubated with previously adherent H9c2 cells for 1 hour, with the addition of hEV^CT^ or hEV^AMI^. Simulated ischaemia was performed for 1 hour. F‐actin was stained with Rhodamine‐Phalloidin (red), and nuclei were stained with DAPI (blue). Scale bars 20 μm. Graph depicts the number of adherent macrophages. *P < 0.05 vs CT, ^#^ *P* < 0.05 vs EV^CT^ (n = 2‐5). (B) Calcein‐AM‐labelled macrophages (green) were added on top of a fibronectin‐based matrix, in the presence of hEV^CT^ or hEV^AMI^. Simulated ischaemia was performed for 1 hour. Nuclei were stained with DAPI (insets; blue). Scale bars 20 μm. Graph depicts the number of adherent macrophages. *P < 0.05 vs CT (n = 2‐5). (C) Macrophages were incubated with opsonized latex beads for 20 minutes (ratio of 10 beads/cell). Non‐internalized beads were labelled with Alexa Fluor 488 antibodies (green). F‐actin was stained with Rhodamine‐Phalloidin (red). Graphs depict the total number of beads/phagocytic macrophages, represented as percentage of fold change over control (CT), the number of beads within cup‐shaped unsealed nascent phagosomes (positive Alexa Fluor 488 staining, arrow heads) and the number of beads in sealed phagosomes (negative Alexa Fluor 488 staining, arrows). Nuclei were stained with DAPI (blue). Scale bars 10 μm. *P < 0.05 vs CT, ^#^ *P* < 0.05 vs EV^CT^ (n = 3‐6)

As with cardiomyocyte‐derived EVs, macrophages incubated with hEV^AMI^ presented higher levels of VEGF, when compared with hEV^CT^ or naïve macrophages (Figure 6F). Moreover, the number of macrophages adhered to cardiomyocytes was lower after incubation with hEV^AMI^, when compared with hEV^CT^ (Figure 7A), whereas challenging with hEV^AMI^ resulted in increased number of macrophages adhered to a fibronectin‐based matrix (Figure 7B). Finally, results in Figure 7C demonstrate that hEV^CT^ enhanced the total number of beads phagocytosed by macrophages, as well as the number of beads in late phagocytic stages, comparing with naïve macrophages. When compared with control EVs, hEV^AMI^ reduced the total number of phagocytosed beads.

## 4 DISCUSSION

In the present work, we explored the role of EV‐mediated communication between cardiomyocytes and macrophages in physiological and pathological conditions, namely in the context of ischaemia, demonstrating for the first time that cardiomyocyte‐derived EVs modulate the phenotype and function of macrophages.

In this study, we show that H9c2‐derived EVs, either from control (EV^CT^) or ischaemia (EV^ISCH^), increased the expression of IL‐1β and IL‐6 in macrophages. This is in accordance with a recent work, which demonstrated that mouse cardiac‐resident macrophages display a distinct gene expression profile, comparing with their spleen and brain counterparts, including constitutive expression of IL‐1β, IL‐6 and IL‐10.^12^ Although requiring further validation, our results suggest that EVs constitutively released by cardiomyocytes play a role in maintaining the specialized profile of cardiac macrophages, including a basal p38MAPK and NF‐κB activation, iNOS, IL‐1β and IL‐6 expression. Moreover, this is in line with the previous reports showing that cardiac macrophages are not overtly polarized in the healthy myocardium, where they present a primarily phagocytic role.^39^

Intriguingly, when comparing the effects of EVs secreted by either H9c2 or NRVM on macrophages, the results were not entirely similar. Given that the immature heart is more resistant to the ischaemic injury than the adult myocardium, it is conceivable that ischaemia differentially impacts the content and/or function of EVs derived from H9c2 or NRVM, ultimately reflecting upon the EV‐triggered effects on macrophages. Moreover, cardiac regeneration in neonatal mice has been proposed to depend on macrophage activation, whose extent and kinetics are altered in older animals.^14^ These effects are concomitant with the terminal differentiation of cardiomyocytes, suggesting that a fine‐tuned exchange of signals between cardiomyocytes and macrophages contributes to the cardiac regenerative ability during this period.^14^ Hence, we can speculate that NRVM‐derived EVs secreted during ischaemia contribute to maintain the specialized profile of macrophages, required to promote regeneration and angiogenesis. On the other hand, in conditions of compromised regenerative capacity, EV^ISCH^ dampen the activation of macrophages, as we show with the EVs isolated from immortalized H9c2 cells, likely contributing to fibrosis and repair of the damaged tissue.

When macrophages were challenged with circulating human EVs, we observed no differences between hEV^CT^ and hEV^AMI^. However, these results should be interpreted with caution as circulating EVs originate from a wide variety of cell types, besides cardiomyocytes, likely explaining the differences found among the effects of H9c2 and human EVs. The presence of contaminating lipoproteins in the EV pellet is another aspect to take into account, since these molecules may contribute to macrophage activation. Notably, the control individuals enrolled in this study presented a high cardiovascular risk, including diabetes and dyslipidaemia, which can affect EV content, ultimately impacting macrophage function. In fact, mounting evidence has suggested that a precise balance of pro‐ and anti‐inflammatory signals is crucial for cardiac repair following ischaemia, which can be disrupted by comorbidities.^40^ Nonetheless, the finding that hEV polarize macrophages towards a non‐canonical phenotype further stresses the importance of EVs in modulating the biology and function of macrophages.

We show that EV^ISCH^, NRVM_EV^ISCH^ and hEV^AMI^ increased VEGF levels in macrophages, supporting the hypothesis that these immune cells play an important proangiogenic role during ischaemia. In fact, previous works reported that macrophage release of VEGF and TGF‐β is critical to preserve cardiac contraction and healing after myocardial infarction.^41^ Nonetheless, according to a recent study by de Couto et al., reporting that EVs secreted by cardiosphere‐derived cells exert protective effects against *I*/*R*, we provide evidence that EV^CT^ are able to protect cardiomyocytes from *I*/*R*‐induced oxidative stress. However, additional in vivo data are required to establish the overall effect of EVs released by cardiomyocytes during ischaemia on disease outcome.

Besides cell‐cell communication, Cx43 channel docking mediates cell adhesion, through a mechanism independent of the GJ‐channel activity.^35^, ^42^ It is conceivable that a disruption of the orchestrated balance between post‐translational modifications of Cx43 during ischaemia, alter not only the channel gating properties, but also affect channel structure, affecting Cx43‐mediated macrophage adhesion to cardiomyocytes.^43^ Recently, it was demonstrated that conditional deletion of macrophage Cx43 delays AV conduction, implicating macrophages in conduction abnormalities, as the ones associated with myocardial ischaemia.^15^ In accordance, our results suggest that EV^CT^ are important to underpin such mechanism. During ischaemia, it is plausible that a decreased adhesion of macrophages to cardiomyocytes in response to EV^ISCH^ contribute and/or exacerbate ischaemia‐induced arrhythmias.

Although heart resident macrophages are crucial to ensure homeostasis, during myocardial ischaemia, this resident population decreases in number, being outgrown by macrophages derived from blood monocytes.^13^, ^44^, ^45^ Therefore, it is possible that a decreased macrophage‐cardiomyocyte adhesion induced by EV^ISCH^ contribute to the depletion of tissue resident macrophages during ischaemia and/or to the macrophage efflux from the inflammation site to draining lymph nodes.^46^ However, one should keep in mind that heart macrophages are a heterogeneous cell population derived from distinct ontological origins.^13^ Therefore, further studies are required to characterize the differential roles of resident and recruited macrophages, as well as the signals involved in their regulation, including those conveyed by EVs.

During the inflammatory phase, a provisional matrix of fibrin and plasma‐derived fibronectin is formed, providing hemostasis and serving as a scaffold for migration and proliferation of leukocytes, endothelial cells and fibroblasts.^36^, ^47^ Accordingly, our results show that EV^ISCH^, NRVM_EV^ISCH^ and hEV^AMI^ promoted the adhesion of macrophages to a fibronectin‐based matrix, which can be particularly important during this repair phase.

When macrophages phagocytize apoptotic cells, they secrete anti‐inflammatory cytokines that actively suppress inflammation, which constitutes an important event in the transition from the inflammatory to the proliferative phase of repair after ischaemia.^11^, ^41^ Although EV^CT^, NRVM_EV^CT^ and hEV^CT^ promote phagocytosis, along with other results obtained in this study, we suggest that impaired and/or slower response elicited by EV^ISCH^ in macrophages precludes their ability to stimulate phagocytosis, comparing with control EVs.

In conclusion, the results presented in this work support a model where EVs released by cardiomyocytes are able to regulate macrophage function.^48^ In basal conditions, cardiomyocytes secrete EVs that induce the polarization of macrophages into a specialized profile, essential for heart homeostasis. During ischaemia, cardiomyocyte‐macrophage crosstalk is affected, with direct implications in the macrophage protective properties likely impairing healing and contributing to adverse cardiac remodelling.

## AUTHOR CONTRIBUTIONS

RAP and TMM performed the experiments and contributed to data analysis. KJ, TMRR, MZ and AS contributed to data acquisition. LR and MS coordinated the collection of human blood samples. PP, PV, JPGS and LG contributed to the writing and revision of the manuscript. MTC contributed to conception and design, data analysis and manuscript writing. HG coordinated the study, designed the concept, analysed data and wrote the manuscript. All authors gave final approval.

## CONFLICT OF INTEREST

The authors declared that there is no conflict of interest.
