# Supplementary material for: Ischaemia alters the effects of cardiomyocyte‐derived extracellular vesicles on macrophage activation
Source: J Cell Mol Med. 2018 Dec 4;23(2):1137–51. doi: 10.1111/jcmm.14014 (PMC6349194; doi:10.1111/jcmm.14014)
Supplement: Supplementary file 1 [file JCMM-23-1137-s001.pdf]

Supplementary Data from *"Ischemia alters the effects of cardiomyocyte-derived extracellular vesicles on macrophage activation"*

Rafael Almeida Paiva[1,2]*, Tania Martins-Marques[1,2]*, Katia Jesus[1,2], Teresa Ribeiro-Rodrigues[1,2], Monica Zuzarte[1,2], Ana Silva[2,3], Liliana Reis[4], Maria da Silva[4], Paulo Pereira[5], Pieter Vader[6,7], Joost Petrus Gerardus Sluijter[8,9], Lino Gonçalves[1,4], Maria Teresa Cruz[2, 3], Henrique Girao[1,2#]

Supplementary Material and Methods

Chemicals

Inhibitor of p38MAPK activity – SB203580 – was obtained from Cell Signaling Technology (Danvers, MA, USA), and endocytosis inhibitor – dynasore – was from Sigma-Aldrich (St. Louis, MO, USA).

Isolation of Neonatal Rat Ventricular Myocytes (NRVM)

NRVM were isolated from Wistar rats, obtained from our local breeding colony. Animals were handled according to European Union guidelines (2010/63/EU), approved by ORBEA-IBILI (permit 13/2015). Briefly, hearts excised from neonatal rats (P3-P5) were subjected to 0.1% trypsin-EDTA digestion overnight, at 4ºC. Type II collagenase (Gibco, 75 U/mL) digestion was further performed for 30 min, at 37ºC, followed by mechanical dissociation of the tissue, and enzyme inactivation by the addition of DMEM containing 10% FBS. Digested tissues were transferred through a screen (70 μm) and cells were recovered by centrifugation and plated into 1% (w/v) gelatin-coated dishes for 3 h. After that, non-adherent cells (enriched in cardiomyocytes) were plated in fibronectin-coated dishes and maintained in DMEM, supplemented with 10% FBS,

1% Penicillin/Streptomycin (100 U/mL:100 µg/mL), at 37ºC, under 5% CO_2, for 48 h before experiments were performed.

Isolation of mouse peritoneal macrophages

Peritoneal macrophages were isolated from C57BL/6 mice, obtained from our local breeding colony. Animals were handled according to European Union guidelines (2010/63/EU), approved by ORBEA-IBILI (permit 13/2015). Mice were anesthetized with isoflurane, after which peritoneal macrophages were obtained by peritoneal lavage, as previously described [1] . Briefly, ice-cold PBS was injected into the peritoneal cavity of mice. The abdominal region was massaged, after which peritoneal fluids were collected. Cells were recovered by centrifugation at 150 *x g*, for 10 min, and plated on cell culture dishes in DMEM, supplemented with 10% FBS, 1% Penicillin/Streptomycin (100 U/mL:100 µg/mL), at 37ºC, under 5% CO_2, for 2 h, to allow purification of macrophages. Non-adherent cells were removed by washing three times with warm PBS.

Cell cultures and simulated ischemia and ischemia-reperfusion (I/R)

Cardiomyoblast cell line H9c2 and macrophage cell line Raw 264.7 (American Type Culture Collection, ATCC TIB-71) were cultured in Dulbecco's Modified Eagle Medium (DMEM, Life Technologies, Carlsbad, CA, USA), supplemented with 10% FBS (no heat inactivation for Raw 264.7 cells), 1% Penicillin/Streptomycin (100 U/mL:100 µg/mL), at 37ºC, under 5% CO_2.

Ischemia was simulated by incubation in an ischemia-mimetic solution (118mM NaCl, 4.7 mM KCl, 1.2 mM KH_2PO_4, 1.2 mM $MgSO_4$, 1.2 mM $CaCl_2$, 25mM $NaHCO_3$, 5 mM lactate, 20 mM 2-deoxy-D-glucose, 20 mM Na-HEPES, pH 6.6), placing the cell-containing dishes in hypoxic pouches (GasPakTM EZ; BD Biosciences, Franklin Lakes, NJ, USA), equilibrated with

95%N_2/5%CO_2 [2] . In reperfusion experiments, after 1 h of ischemia, ischemia-mimetic solution was replaced by complete medium, and cells returned to normoxia.

EV isolation from human serum samples

Venous blood samples were collected into a non-heparinized tube (BD Vacutainer SST II Plus plastic serum tube, BD Biosciences) within 12 hours after admission to the Coronary Intensive Care Unit, in the case of AMI patients, or within 20 minutes after coronary angiography in the controls. Blood was allowed to clot at room temperature (RT) for approximately 30 min, after which serum was retrieved in the supernatant by centrifugation at 1.000 x *g* for 15 min at RT. Serum samples were diluted in PBS (1:2), centrifuged at 2.000 x *g* for 30 min, followed by 45 min at 12.500 x *g*. Supernatants were ultracentrifuged for 2h at 100.000 x *g*, after which pellets were resuspended in PBS, filtered (0.22 μm filter) and further ultracentrifuged at 100.000 x *g*, for 70 min. A last wash with PBS was performed, followed by ultracentrifugation at 100.000 x *g*, for 70 min. Final pellets were resuspended in sterile PBS for further experiments. Total protein content of EVs was determined using the BCA Protein Assay Reagent Kit (Thermo Fisher Scientific, Waltham, MA, USA). On average, we obtained 45.7 μg/mL serum of human controls, and 46.7 μg/mL serum of AMI patients.

Transmission electron microscopy (TEM)

EVs were fixed with 2% paraformaldehyde (PFA) and deposited on Formvar-carbon coated grids (TAAB Laboratories Equipment, Berks, UK). Samples were washed with PBS and fixed with 1% glutaraldehyde for 5 min. Grids were washed with water, contrasted with an uranyl-oxalate solution pH 7, for 5 min, and transferred to methyl-cellulose – uranyl acetate for 10 min on ice, as previously

described [3, 4] . Images were collected using a Tecnai G2 Spirit BioTWIN electron microscope (FEI, Oregon, USA) at 80kV.

Nanoparticle tracking analysis (NTA)

EVs were ressuspended in 1 ml of PBS, after which NTA was performed using NanoSight LM 10 instrument (NanoSight Ltd). Analysis settings were optimized and kept constant between samples and each video was analyzed to give the mean size and estimated concentration of particles. Data were processed using NTA 2.2 analytical software.

Western Blot

Cell and EV lysates were prepared for WB analysis as described before [3, 4] . Primary antibodies against Cx43, GAPDH, Calnexin (Sicgen, Cantanhede, Portugal), phosphorylated/total p38-MAPK and phosphorylated/total NF-κB/p65 (Cell Signaling Technologies), VEGF (abcam, Cambridge, UK), Tubulin (Sigma-Aldrich) and iNOS (R&D Systems, Minneapolis, MN, USA) were used, followed by horseradish peroxidase (HRP)-conjugated secondary antibodies and visualized by chemiluminescence using a VersaDoc system (BioRad, Hercules, CA, USA). Densitometric quantification was performed in unsaturated images using Image J (National Institutes of Health).

Cell viability assessment

5 mg/mL of 3-(4,5-Dimethylthiazol-2-yl)-2,5-Diphenyltetrazolium Bromide (MTT) solution was added to each well, and incubated at 37ºC, 5% CO_2, for 15 min (in Raw 264.7 cells) or 2 h (in H9c2 cells). Acidic isopropanol (0.04 N HCl) was used to dissolve the formazan crystals. Formazan

quantification was performed using an ELISA automatic microplate reader (SLT, Austria), at 570 nm, with a 620 nm reference wavelength.

Real-time RT-PCR

Total RNA was isolated from cells with TRIzol reagent (Thermo Fisher Scientific), according to the manufacturer's instructions. RNA concentrations were determined using a NanoDrop spectrophotometer (Thermo Fisher Scientific). Total RNA was reverse-transcribed using the iScript Select cDNA synthesis kit (Bio-Rad). Real-time RT-PCR reactions were performed using SYBR® Green Supermix (Bio-Rad) and appropriate primers, in a Bio-Rad MyCycler iQ5. Gene expression changes were analyzed using the built-in iQ5 Optical system software. The primers used were the following:

# primer name	sequence
IL1b FW	ACCTGTCCTGTGTAATGAAAG
IL1b REV	GCTTGTGCTCTGCTTGTG
IL-4 FW	TTAATTGTCTCTCGTCACTG
IL-4 REV	GTTTGGCACATCCATCTC
IL-6 FW	TTCCATCCAGTTGCCTTC
IL-6 REV	TTCTCATTTCCACGATTTCC
IL-10 FW	CCCTTTGCTATGGTGTCCTTTC
IL-10 REV	ATCTCCCTGGTTTCTCTTCCC
IL-12p40 FW	TGTCGTAGAATTGGATTGGTATC
IL-12p40 REV	AACCTCGCCTCCTTTGTG

Arginase FW	GTGCCCTCTGTCTTTTAG
Arginase REV	GCTCCGATAATCTCTAAGG
TNF-alfa FW	CAAGGGACTAGCCAGGAG
TNF-alfa REV	TGCCTCTTCTGCCAGTTC
GAPDH FW	GCCTTCCGTGTTCCTACC
GAPDH REV	GCCTGCTTCACCACCTTC

Immunofluorescence staining

Immunofluorescence staining of cultured cells was performed as previously described [2] . Briefly, cells were fixed with 4%PFA, blocked, permeabilized and stained using primary antibodies against Cx43 (Sicgen), 4-HNE (OXIS International, Beverly Hills, CA, USA) or Troponin T (abcam). Appropriate secondary antibodies were used, together with F-actin staining (Rhodamine-Phalloidin, Sigma-Aldrich), when applicable. For experiments on heart slices, BALB/c mice were used. Animals were obtained from our local breeding colony (permit 13/2015) and handled according to European Union guidelines (2010/63/EU). Mice were sacrificed, after which hearts were harvested and immediately embedded in optimum cutting temperature (OCT) matrix (Tissue-Tek) for cryosectioning (5 μm slices), before storage at -80ºC. Immunofluorescence was performed as described previously [2] . Primary antibodies against Cx43 (Sicgen) and CD11b (abcam) were used. Images were acquired by confocal microscopy, using a Zeiss LSM 710.

Determination of cellular ATP levels

H9c2 were subjected to control or simulated ischemia conditions for 20 min, after which cellular ATP levels were determined, using the Luminescent ATP Detection Assay Kit (abcam). Manufacturer's instructions were followed.

References

[1] **Zhang X, Goncalves R, Mosser DM**. The isolation and characterization of murine macrophages. *Curr. Protoc. Immunol.* 2008; Chapter 14; Unit 14.1.

[2] **Martins-Marques T, Catarino S, Zuzarte M, et al.** Ischaemia-induced autophagy leads to degradation of gap junction protein connexin43 in cardiomyocytes. *Biochem. J.* 2015; 467; 231–45.

[3] **Soares AR, Martins-Marques T, Ribeiro-Rodrigues T, et al.** Gap junctional protein Cx43 is involved in the communication between extracellular vesicles and mammalian cells. *Sci. Rep.* 2015; 5; 1–13.

[4] **Martins-Marques T, Pinho MJ, Zuzarte M, et al.** Presence of Cx43 in extracellular vesicles reduces the cardiotoxicity of the anti-tumour therapeutic approach with doxorubicin. *J. Extracell. Vesicles* 2016; 5.

Supplementary Figures

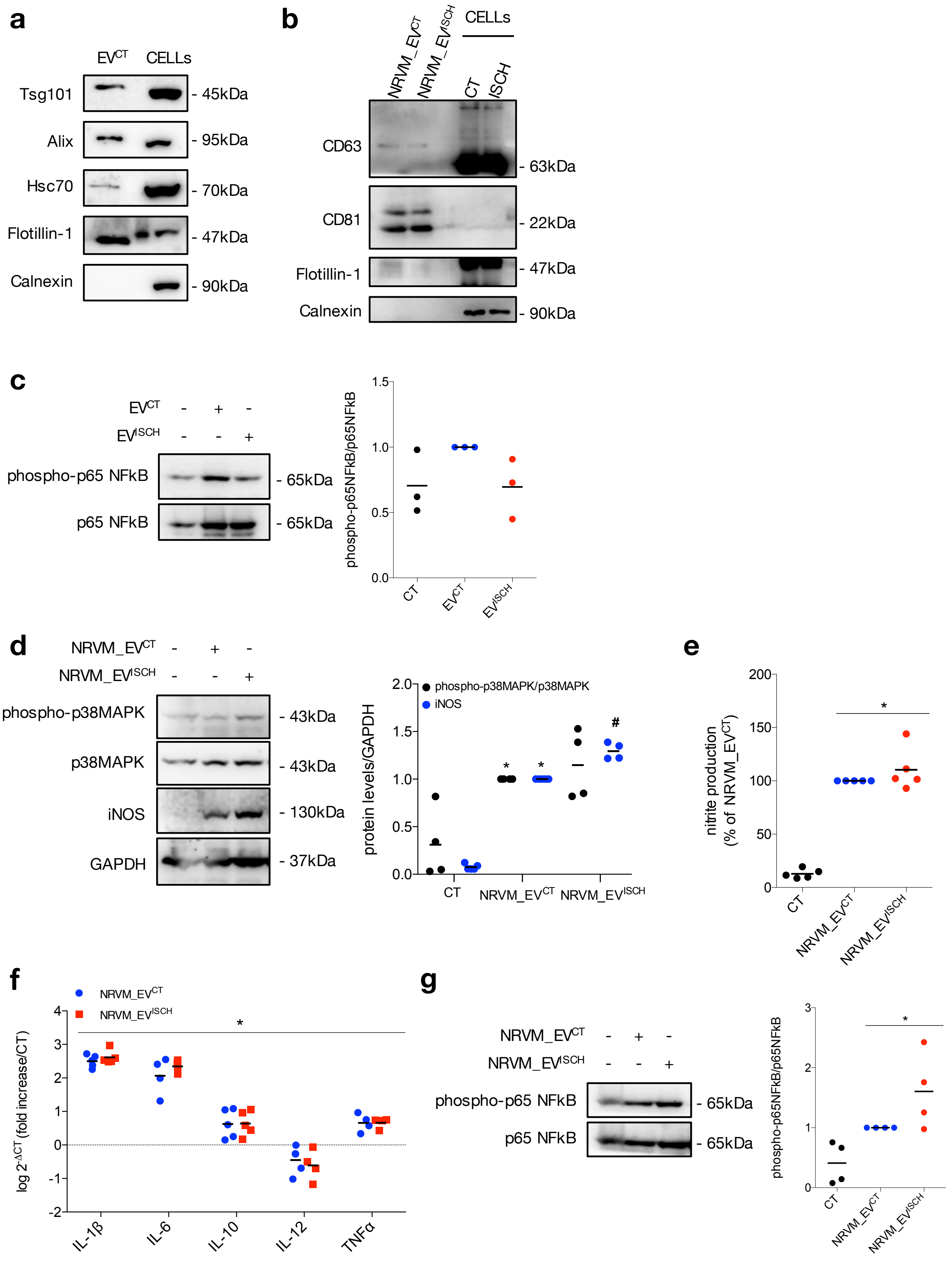

Supplementary Fig. 1

NRVM-derived EVs induce activation of macrophages. a. WB analysis of canonical EV markers – Tsg101, ALG-2-interacting protein X (Alix), Hsc70 and Flotillin-1 – in EVs isolated from H9c2 cells. Cellular extracts were used as control (CELLs). 10 µg of total protein were loaded in each case. **b.** NRVM were cultured under control (NRVM_EV^{CT}) or simulated-ischemia conditions (NRVM_EV^{ISCH}) for 2 h, after which EVs were isolated by differential centrifugation. The protein profile of EVs and cellular extracts was evaluated by WB. 10 µg of total protein were loaded in each case. **c.** Phosphorylation of NF-κB/p65 was evaluated by WB in macrophages after incubation with H9c2-derived EV^{CT} or EV^{ISCH} for 24 h. Graph depicts WB quantification (n=3). **d.** Macrophages were incubated with NRVM_EV^{CT} or NRVM_EV^{ISCH} for 24 h. p38MAPK phosphorylation and iNOS expression were evaluated. Graph depicts WB quantification. *$p<0.05$ vs CT, #$p<0.05$ vs NRVM_EV^{CT} (n=4). **e**. Nitrite production was determined using the Griess reagent assay in macrophages stimulated with NRVM_EV^{CT} or NRVM_EV^{ISCH} for 24 h. Results are expressed as percentage of nitrite production over macrophages treated with NRVM_EV^{CT}. *$p<0.05$ vs CT (n=5). **f.** mRNA expression levels of IL-1β, IL-6, IL-10, IL-12 and TNFα were assessed by RT-qPCR in macrophages stimulated with NRVM_EV^{CT} or NRVM_EV^{ISCH} for 24 h. Results were normalized using GAPDH and expressed relatively to naïve macrophages (CT). Values are expressed as $\log^{2-\Delta CT}$. *$p<0.05$ vs CT (n=5). **g.** Phosphorylation of NF-κB/p65 was evaluated by WB in macrophages after incubation with NRVM_EV^{CT} or NRVM_EV^{ISCH} for 24 h. Graph depicts WB quantification. *$p<0.05$ vs CT (n=4).

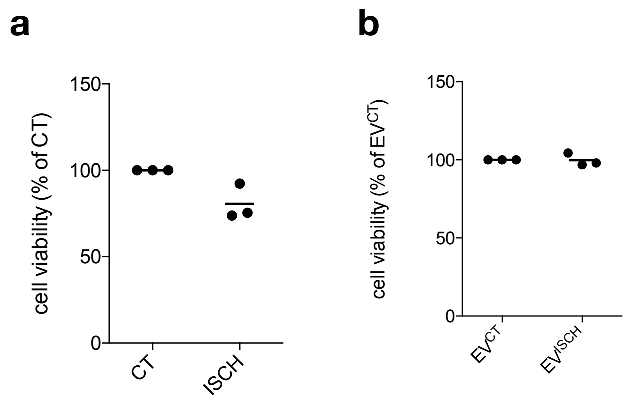

Supplementary Fig. 2

Ischemic cardiomyocyte-released EVs do not affect the viability of macrophages. Cell viability was evaluated using the MTT assay in **(a)** H9c2 cells maintained in control conditions (CT) or subjected to ischemia (ISCH) for 2 h, and **(b)** macrophages stimulated with H9c2-derived EV^{CT} or EV^{ISCH} for 24 h.

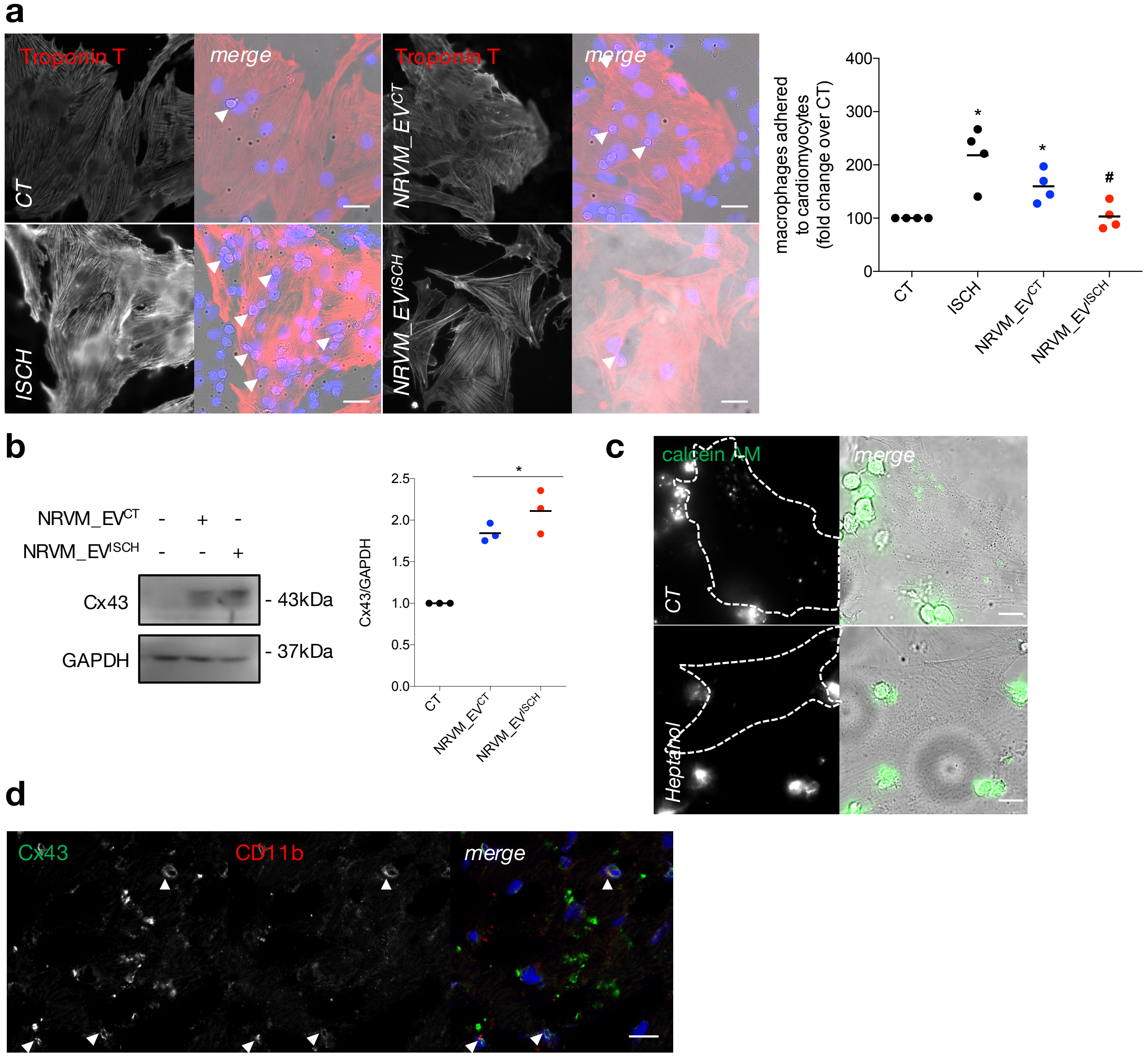

Supplementary Fig. 3

Cx43 staining is found in adhesion contacts between cardiomyocytes and macrophages. a. Macrophage suspension was incubated with previously adherent NRVM culture for 1 h. NRVM_EV^{CT} or NRVM_EV^{ISCH} were added, where indicated. Simulated ischemia was performed for 1 h, by incubation with an ischemia-mimetic buffer under hypoxia. F-actin was stained with Rhodamine-Phalloidin (red), and nuclei were stained with DAPI (blue). Scale bars 20 μm. Graph

depicts the number of adherent macrophages. *p<0.05 vs CT, #p<0.05 vs NRVM_EV^{CT} (n=4). **b.** Cx43 levels were evaluated by WB in macrophages after incubation with NRVM-derived EV^{CT} or EV^{ISCH} for 24 h. Graph depicts WB quantification. *p<0.05 vs CT (n=3) **c.** Macrophages were labelled with 1.5 µM Calcein-AM (green) and further incubated with previously adherent H9c2 cells, for 1 h. Non-fixed cells were immediately observed by fluorescence microscopy. Dashed lines highlight the contours of cardiomyocytes. Scale bars 10 µm (n=2). **d.** Mouse heart slices were immunostained for Cx43 and CD11b. Nuclei were stained with DAPI. Scale bars 20 µm (n=2).

a

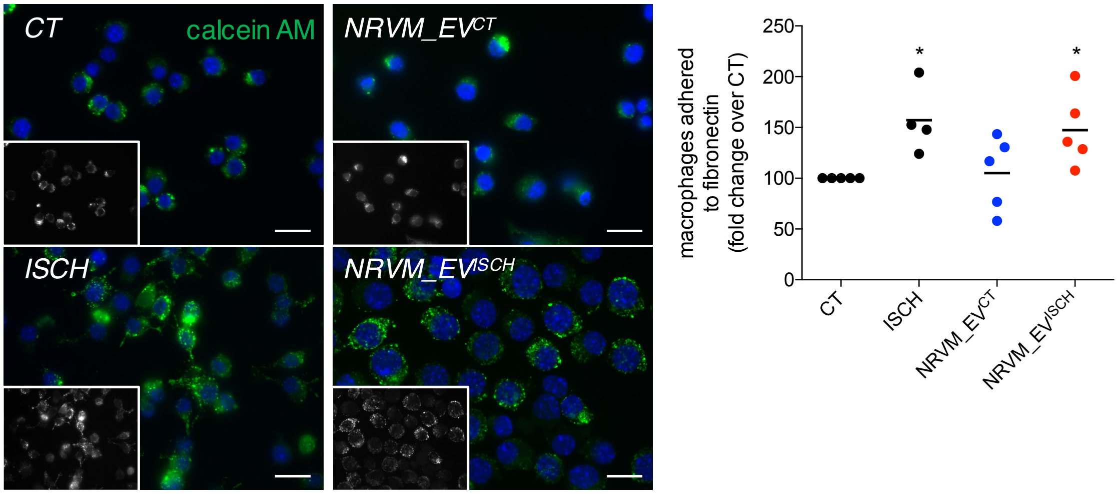
CT
calcein AM
NRVM_EV^CT
ISCH
NRVM_EV^ISCH
macrophages adhered
to fibronectin
(fold change over CT)
250
200
150
100
50
0
*
*
CT
ISCH
NRVM_EV^CT
NRVM_EV^ISCH

b

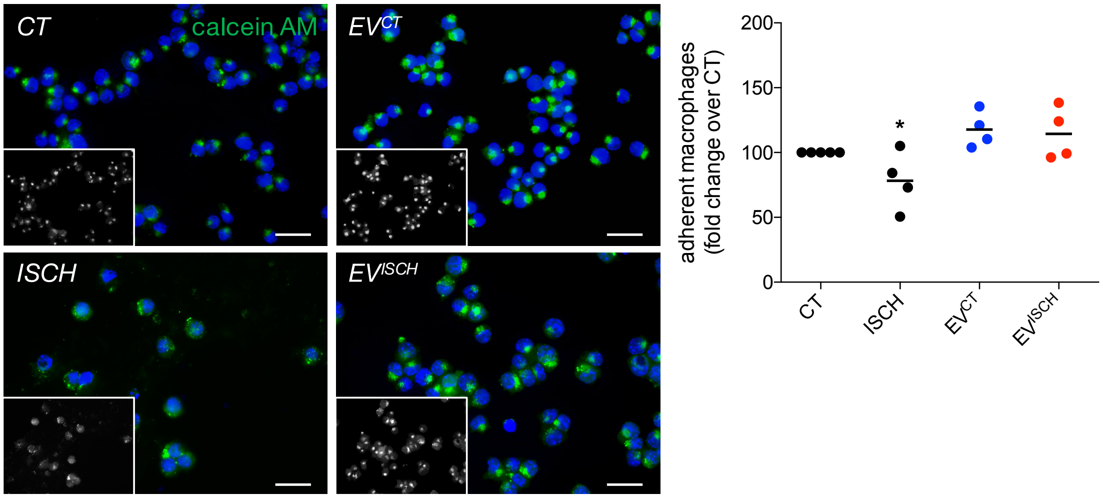
CT
calcein AM
EV^CT
ISCH
EV^ISCH
adherent macrophages
(fold change over CT)
200
150
100
50
0
*
CT
ISCH
EV^CT
EV^ISCH

c

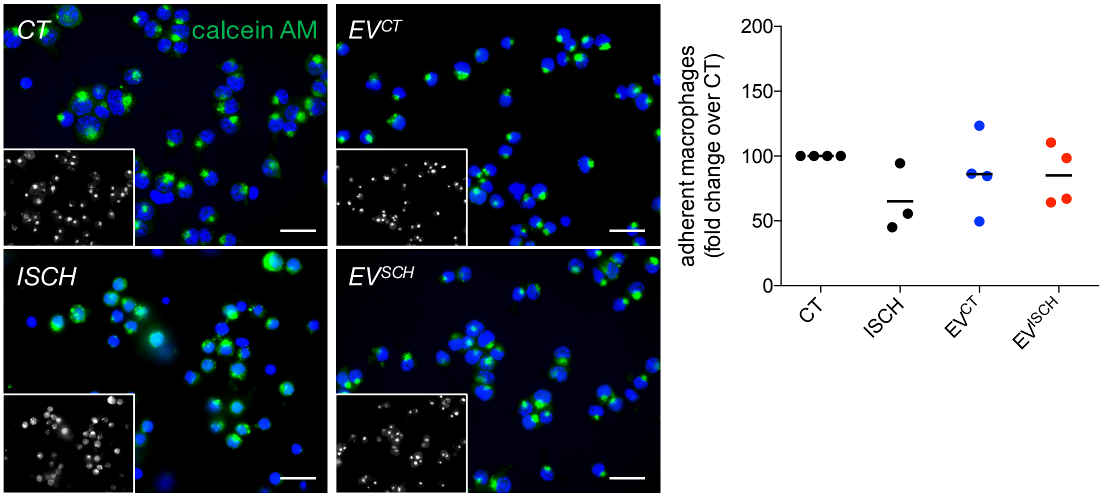
CT
calcein AM
EV^CT
ISCH
EV^SCH
adherent macrophages
(fold change over CT)
200
150
100
50
0
CT
ISCH
EV^CT
EV^ISCH

Supplementary Fig. 4

Cardiomyocyte-derived EVs do not alter macrophage adhesion to collagen-based matrices. **a.** Calcein AM-labeled macrophages (green) were added on top of a fibronectin-based **(a)**, collagen I **(b)** or collagen IV **(c)** matrix. NRVM_EV^{CT} or NRVM_EV^{ISCH} **(a)** or H9c2-derived EV^{CT} or EV^{ISCH} **(b-c)** were added, where indicated. Simulated ischemia was performed for 1h. Nuclei were stained with DAPI (blue). Scale bars 20 μm. Graphs depict the number of adherent macrophages. *$p<0.05$ vs CT (n=4).

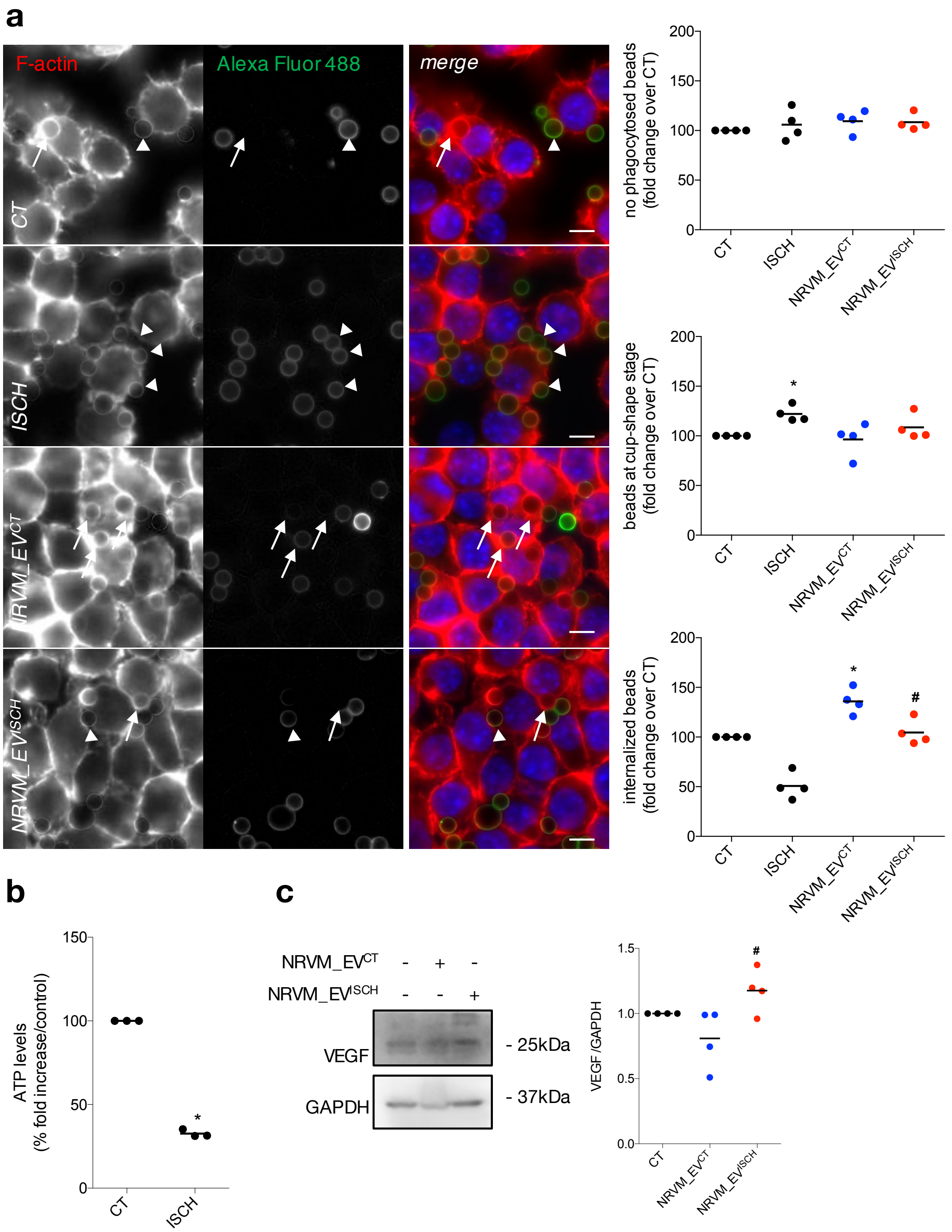

Supplementary Fig. 5

Control NRVM_EVs increase the phagocytic capacity of macrophages. a. Macrophages were incubated with opsonized latex beads for 20 min (ratio of 10 beads/cell). Non-internalized beads were labelled with Alexa Fluor 488 antibodies (green). F-actin was stained with Rhodamine-

Phalloidin (red). Graphs depict the total number of beads/phagocytic macrophages, represented as percentage of fold change over control (CT), the number of beads within cup-shaped unsealed nascent phagosomes (positive Alexa Fluor 488 staining, arrow heads) and the number of beads in sealed phagosomes (negative Alexa Fluor 488 staining, arrows). Nuclei were stained with DAPI (blue). Scale bars 10 μm. *p<0.05 vs CT, #p<0.05 vs NRVM_EV^{CT} (n=4). **b.** H9c2 cells were subjected to simulated ischemia for 20 minutes, after which cellular ATP levels were measured. *p<0.05 vs CT (n=3). **c.** Macrophages were incubated with NRVM_EV^{CT} or NRVM_EV^{ISCH} for 24 h. VEGF levels were analyzed by WB and depicted on graph. #p<0.05 vs NRVM_EV^{CT} (n=4).

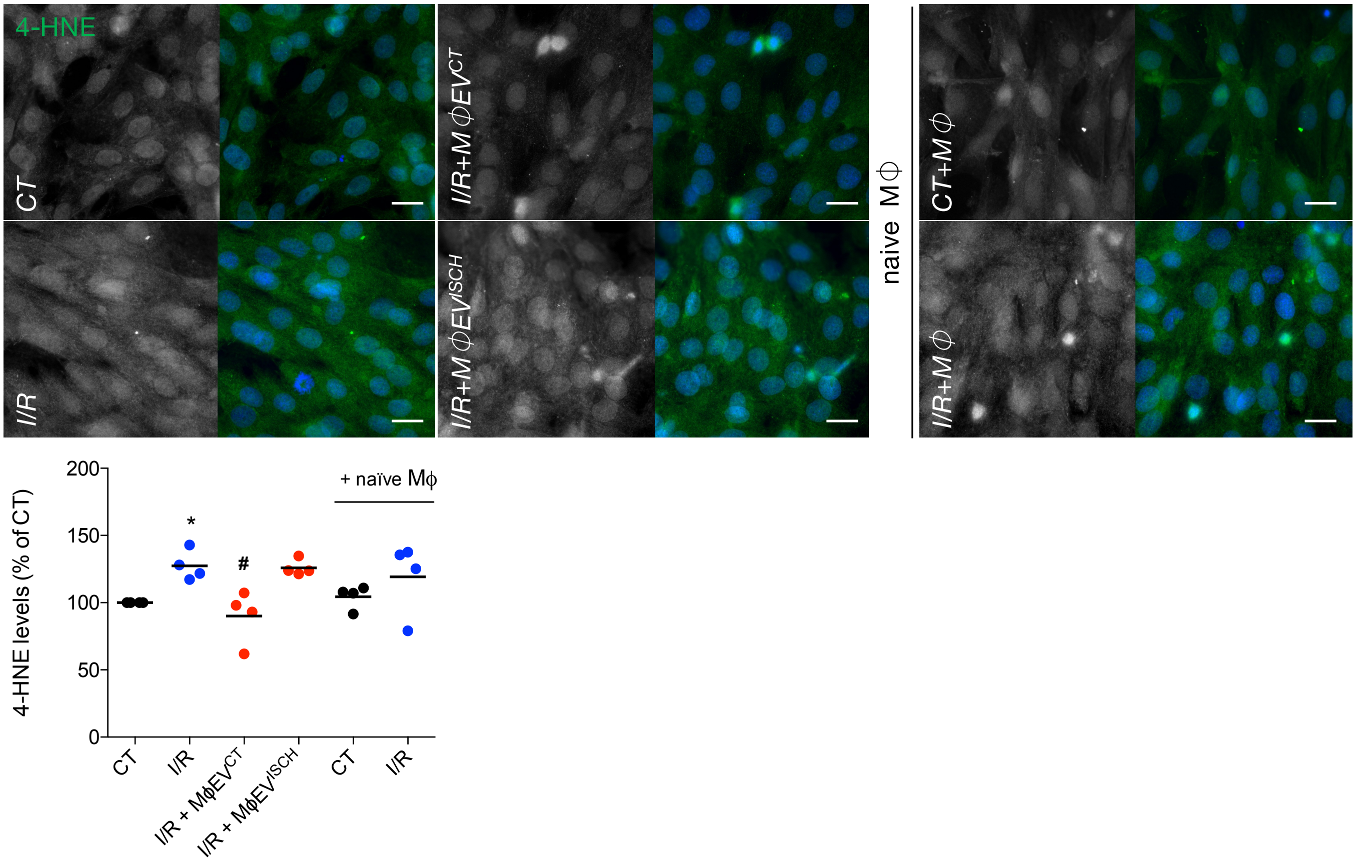

Supplementary Fig. 6

Macrophages stimulated with control EVs protect cardiomyocytes from I/R-induced injury. Macrophages were incubated with H9c2-derived EV^{CT} or EV^{ISCH} for 24 h, after which the cells were scraped and co-cultured with H9c2 cardiomyoblasts. Cells were subjected to 1h of ischemia, followed by 30 min of reperfusion (I/R) in the presence or absence of naïve (Mϕ) or primed macrophages (Mϕ+EV^{CT} or Mϕ+EV^{ISCH}). After I/R, co-cultures were immunostained for 4-HNE (green). Nuclei were stained with DAPI (blue). Scale bars 10 µm. Graph depicts quantification of 4-HNE levels. *p<0.05 vs CT, # p<0.05 vs I/R (n=4).

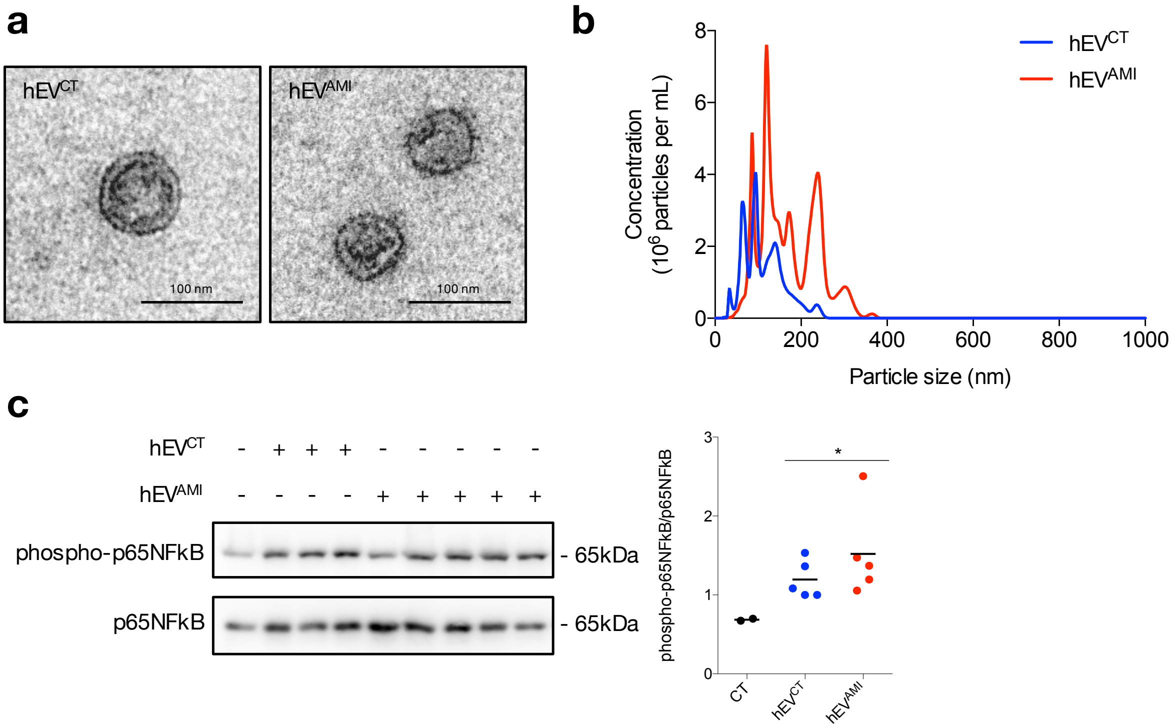

Supplementary Fig. 7

Circulating human EVs induce activation of macrophages. a. Representative TEM images of circulating hEV^{CT} and hEV^{AMI}. **b**. NTA analysis of circulating human EVs (n=2-4). Concentration

indicate the number of particles per ml of serum sample. **c**. Phosphorylation of NF-kB/p65 was evaluated by WB in macrophages after incubation with hEV^{CT} or hEV^{AMI} for 24 h. Graph depicts WB quantification *$p<0.05$ vs CT (n=2-6).
